# TCA cycle rewiring underpins histone acetylation sourcing and cell-fate transitions during exit from naive pluripotency

**DOI:** 10.1016/j.stem.2026.04.004

**Published:** 2026-05-07

**Authors:** Eleni Kafkia, David Pladevall-Morera, Lidia Argemi-Muntadas, Gangqi Wang, Roberta Noberini, Arnau Casòliba-Melich, Sandra Bagés-Arnal, Matthias Anagho-Mattanovich, Rita Silvério-Alves, Johanna Gassler, Tiziana Bonaldi, Ton J. Rabelink, Thomas Moritz, Jan Jakub Żylicz

**Affiliations:** 1 Novo Nordisk Foundation Center for Stem Cell Medicine—reNEW, Department of Biomolecular Sciences, Faculty of Health and Medical Sciences, University of Copenhagen, Copenhagen, Denmark; 2 Novo Nordisk Foundation Center for Basic Metabolic Research, Faculty of Health and Medical Sciences, University of Copenhagen, Copenhagen, Denmark; 3 Department of Internal Medicine (Nephrology) & Einthoven Laboratory of Vascular and Regenerative Medicine, Leiden University Medical Center, Leiden, the Netherlands; 4 Novo Nordisk Foundation Centre for Stem Cell Medicine—reNEW, Leiden University Medical Centre, Leiden, the Netherlands; 5 Department of Experimental Oncology, IEO, European Institute of Oncology IRCCS, 20139 Milan, Italy; 6 Department of Oncology and Hematology-Oncology, University of Milano, 20122 Milano, Italy

**Keywords:** metabolism, development, embryo, stem cells, epigenetics, spatial metabolomics, 13C isotope tracing, histone acetylation, pluripotency, differentiation

## Abstract

Metabolism shapes stem cell differentiation and epigenome regulation, especially during the exit from naive pluripotency *in vitro*. Yet how metabolic networks reorganize at implantation remains unclear. Here, we map metabolite routing in pre- and post-implantation mouse embryos and across dynamic pluripotency transitions in stem cells, revealing that the tricarboxylic acid (TCA) cycle undergoes spatio-temporal rewiring rather than a simple shutdown. Pyruvate emerges as a central metabolic nexus, where pyruvate carboxylase and malic enzyme activities create a cyclical carbon flow essential for balanced metabolic and transcriptional states, timely exit from naive pluripotency, and differentiation. As cells leave naive pluripotency, glutamine increasingly fuels the TCA cycle; unexpectedly, it is also the dominant carbon source for histone acetylation. The necessary acetyl-CoA is generated via IDH1-mediated reductive glutamine carboxylation and is coupled to pyruvate cycling, sustaining histone acetylation. These findings uncover a metabolically rewired, route-specific nutrient utilization program that links metabolism to epigenomic regulation and pluripotency transitions at implantation.

## Highlights

• TCA cycle rewiring underpins embryo implantation and exit from naive pluripotency
• Pyruvate cycling enables a timely exit from naive pluripotency and differentiation
• Glutamine is the primary metabolic source for histone acetylation in pluripotency
• Pyruvate cycling and reductive glutamine carboxylation sustain histone acetylation

## Graphical abstract

**Figure:**
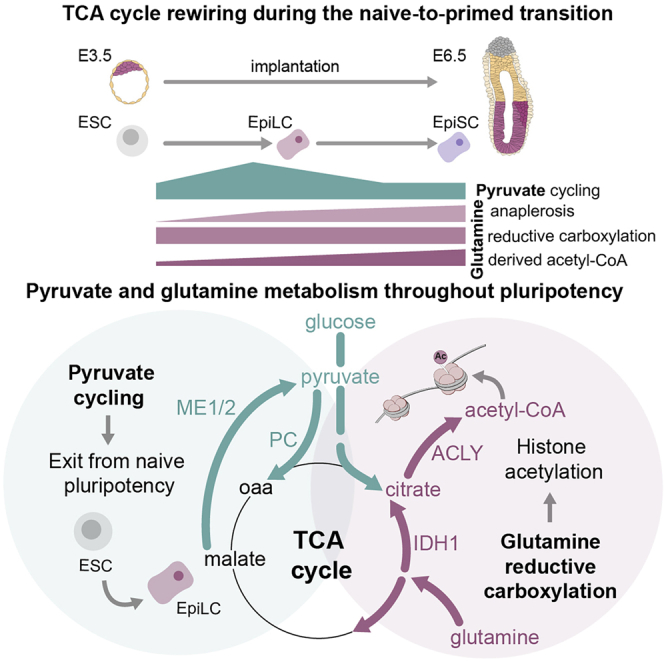

## Teaser

Using carbon tracing and functional experiments, Kafkia, Pladevall-Morera, et al. show that TCA cycle rewiring underlies mouse embryo implantation and the exit from naive pluripotency. In this context, pyruvate cycling enables timely cell-state transitions, while, coupled to it, reductive glutamine metabolism fuels the histone acetylation backbone.

## Introduction

Early mammalian development, and implantation in particular, involves dramatic transcriptional, epigenetic, and metabolic programming, which coordinate successful pregnancy. Similarly to other modalities, metabolism also operates as a regulatory pathway influencing cell-state and cell-fate transitions via its coupling to the epigenome and signaling.^1^^,^^2^^,^^3^ Following fertilization in mice, there is rapid metabolic rewiring, which culminates when the blastocyst exhibits a bivalent metabolic state.^4^^,^^5^^,^^6^^,^^7^ Specifically, the blastocyst uptakes glucose and uses glycolysis intermediates for biosynthetic processes, while funneling pyruvate and lactate into the tricarboxylic acid (TCA) cycle, thereby fueling oxidative phosphorylation (OxPhos) for energy production.^8^^,^^9^ How metabolism is wired at later developmental stages remains rather elusive. The implanted conceptus exists in a highly oxygen-deprived environment, robustly consuming glucose, suggesting reduced OxPhos activity and increased reliance on glycolysis.^8^^,^^9^^,^^10^ Later, at E6.75–7.5 during primitive streak formation, glucose metabolism becomes coupled to somatic lineage allocation.^11^ Nevertheless, direct evidence for how metabolism is rewired at implantation is lacking.

Implantation is accompanied by a continuum of cell-state transitions that can be modeled *in vitro* by different pluripotent stem cells (PSCs), namely embryonic stem cells (ESCs), epiblast-like cells (EpiLCs), and epiblast stem cells (EpiSCs), representing naive, formative, and primed pluripotency, respectively.^12^^,^^13^^,^^14^^,^^15^^,^^16^ By and large, the transition from bivalent to exclusively glycolytic metabolism seen in naive ESCs versus primed EpiSCs^17^ mirrors predictions for metabolic changes in the peri-implantation embryo.^8^^,^^9^ On a functional level, α-ketoglutarate (αKG) and glutamine availability are important modulators of naive pluripotency.^18^^,^^19^^,^^20^ Additionally, ESCs show unanticipated complexity in how metabolic pathways operate, as they engage a non-canonical fragmented TCA cycle reliant on ATP-citrate lyase (ACLY).^21^ Despite these insights, the dynamic rewiring of metabolism during the exit from naive pluripotency and its fidelity with what occurs in the embryo remain unresolved, especially given that prior studies relied on high, atmospheric oxygen levels.

Chromatin has emerged as one of the sensors of the intracellular metabolic state.^1^^,^^2^ Implantation constitutes the most dramatic stage of accumulating somatic epigenetic memory in the form of repressive histone and DNA methylation.^22^^,^^23^^,^^24^ This coincides with rapid enhancer and promoter switching that programs a new histone acetylation profile,^25^ a feature that is crucial for successful development.^26^^,^^27^^,^^28^^,^^29^^,^^30^^,^^31^ Depositing such activating chromatin modifications by acetyltransferases relies on the availability of acetyl-CoA.^32^^,^^33^ Nevertheless, the mechanisms by which PSCs sustain adequate bioenergetic and biosynthetic functions under low oxygen, while concurrently maintaining and repositioning histone acetylation, remain poorly understood.

**Figure 1:**
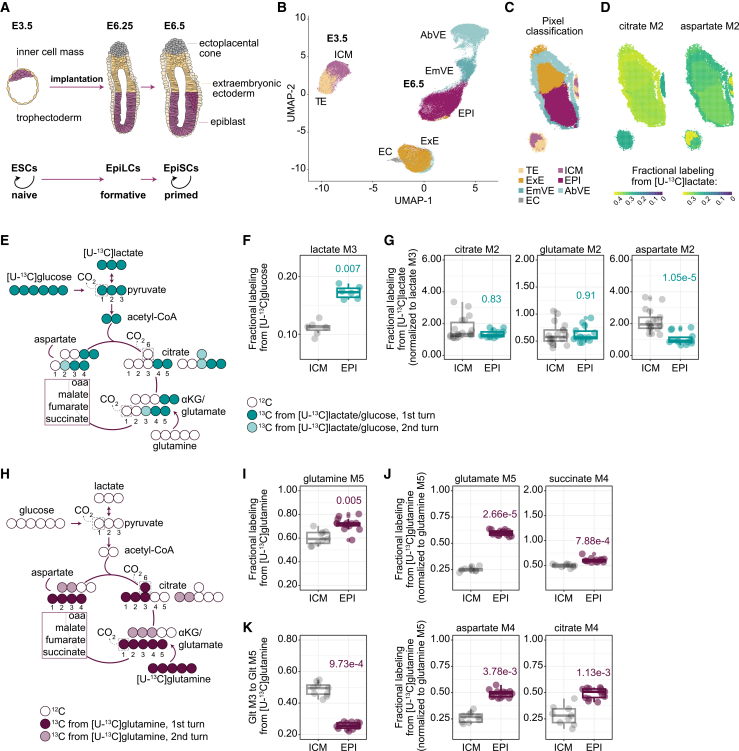
Spatially programmed TCA rewiring underpins embryo implantation (A) Schematic of the embryonic stages and *in vitro* models used. (B and C) UMAP analysis (B) and spatial segmentation (C) of E3.5 and E6.5 embryos based on lipid signals. ICM, inner cell mass; TE, trophectoderm; EPI, epiblast; EmVE, embryonic visceral endoderm; AbVE, abembryonic visceral endoderm; ExE, extraembryonic ectoderm; EC, ectoplacental cone. (D) Spatial distribution of M2 citrate and M2 aspartate derived from the [U-^13^C]lactate in E3.5 and E6.5 embryos. (E) Schematic of carbon transitions in the TCA cycle using [U-^13^C]glucose or [U-^13^C]lactate tracers. ^13^C carbons are colored, with the lighter shade indicating a second TCA cycle turn, and unlabeled ^12^C carbons are shown as empty. (F) Fractional labeling of M3 lactate from [U-^13^C]glucose (ICM, *n* = 7; EPI, *n* = 6). (G) Fractional labeling of M2 citrate, glutamate, and aspartate from [U-^13^C]lactate normalized to M3 lactate (ICM, *n* = 18; EPI, *n* = 14). (H) Schematic of carbon transitions in the TCA cycle using a [U-^13^C]glutamine tracer. Carbons are colored as in (E). (I) Fractional labeling of M5 glutamine from [U-^13^C]glutamine (ICM, *n* = 9; EPI, *n* = 14). (J) Fractional labeling M5 glutamate, M4 succinate, M4 aspartate, and M4 citrate from [U-^13^C]glutamine normalized to M5 glutamine (ICM, *n* = 9; EPI, *n* = 14). (K) Ratio of M3 glutamate to M5 glutamate from [U-^13^C]glutamine (ICM, *n* = 9; EPI, *n* = 14). (F, G, and I–K) Each point represents one embryo. Boxplots depict the median and interquartile range, with whiskers indicating the minimum and maximum values. Statistics were calculated using the Kruskal-Wallis test with Dunn’s post hoc test, with and Benjamini-Hochberg adjustment of *p* values. See also Figures S1 and S2; Table S1.

Here, we sought to define how central carbon metabolism is dynamically reorganized at implantation in mouse embryos and across pluripotency transitions in PSCs (Figure 1A). To this end, we combined ^13^C isotope tracing under physiological oxygen conditions with spatially resolved matrix-assisted laser desorption/ionization mass spectrometry imaging (MALDI-MSI). We reveal that, rather than a simple shutdown, there is an intricate spatio-temporal TCA cycle rewiring as cells exit naive pluripotency. Pyruvate emerges as a key metabolic node, with pyruvate carboxylase (PC) and malic enzyme (ME) activities establishing a cyclical carbon flow that maintains the metabolic and transcriptional state of ESCs and enables timely exit from naive pluripotency and differentiation. Across the pluripotency spectrum, there is robust reverse carbon flow through reductive glutamine metabolism, which in formative and primed pluripotency states is supported by enhanced glutamine anaplerosis. Strikingly, this sustains epigenome programming, as isocitrate dehydrogenase 1 (IDH1)-dependent, glutamine-derived acetyl-CoA preferentially fuels the histone acetylation backbone, highlighting that glucose is not the dominant carbon source for this process. In this context, pyruvate cycling and reductive glutamine carboxylation functionally cooperate to sustain histone acetylation. Together, our study reveals unexpected complexity in metabolic rewiring at implantation and exit from naive pluripotency. These diverse nutrient usage strategies are essential for maintaining developmental progression and converge on sustaining rapid epigenome remodeling.

## Results

### Spatially programmed TCA rewiring underpins embryo implantation

To uncover how metabolism rewires in the embryo as the naive epiblast (EPI) becomes primed for gastrulation, we adapted a spatial metabolomics method combining MALDI-MSI with stable isotope tracing for mouse embryos.^34^^,^^35^ Isolated pre-implantation E3.25 blastocysts and post-implantation E6.25 embryos (Figure 1A) were cultured for 5 h under 5% O_2_ in chemically defined media supplemented with [U-^13^C]glucose, [U-^13^C]lactate, or [U-^13^C]glutamine, representing key carbon sources for the embryo. Spatial lipid distributions at 5-μm resolution were visualized on a uniform manifold approximation and projection (UMAP) plot (Figure 1B). Seurat clusters of these pixels were then annotated to individual lineages and stages based on their stereotypical location (Figure 1C). The E3.5 embryo included the inner cell mass (ICM) and trophectoderm (TE), whereas E6.5 included the EPI, embryonic visceral endoderm (EmVE), abembryonic VE (AbVE), extraembryonic ectoderm (ExE), and ectoplacental cone (EC) (Figure 1C). Next, signals from pixels originating from the same embryo and cluster were averaged and further quantified. Overall, lipids separated known cell types regardless of the ^13^C tracer (Figure 1C). Using this lipidomic fingerprint, we tracked the incorporation of ^13^C carbons in glycolytic and TCA cycle intermediates across distinct lineages (Figure 1D). αKG and oxaloacetate labeling was approximated by the labeling patterns of glutamate and aspartate, respectively, given their rapid equilibration through transamination reactions.^36^ In this context, [U-^13^C]glucose generates pyruvate and lactate labeled in all three carbons (M3 isotopologues), and pyruvate dehydrogenase complex (PDH)-mediated TCA entry yields metabolites carrying two ^13^C carbons (M2 isotopologues) after one round (Figure 1E). Tracing [U-^13^C]glucose revealed that E6.5 embryos produce more lactate than E3.5 (Figures 1F and S1A), indicating higher glycolytic activity upon implantation. [U-^13^C]lactate tracing showed increased lactate uptake at E6.5 embryos compared with E3.5 (Figure S1B), and that lactate was further oxidized in the TCA cycle similar to glucose (Figure S1B). M2 citrate increased at E6.5 relative to E3.5 embryos (Figure S1B), but this difference was lost after normalization to M3 lactate, suggesting comparable lactate contribution to TCA cycle entry between pre- and post-implantation embryos (Figures 1G and S2B). The same pattern was observed for glutamate (Figure 1G). In contrast, aspartate, a metabolite further downstream in the TCA cycle, showed decreased labeling in E6.5 compared with E3.5 embryos (Figures 1G, S1A, S1B, S2A, and S2B). This suggested a relative increase in dilution from unlabeled sources downstream of αKG/glutamate and/or altered TCA cycling (oxidative activity). Interestingly, M2 aspartate differed between E3.5 lineages, with ICM showing higher percentage of labeling than TE (Figures 1D, S1A, S1B, S2A, and S2B). Additional analysis considering per-embryo variability identified further lineage-specific signatures (Figure S2F). Together, these findings revealed both spatial and temporal control of metabolic flows in the developing mouse embryo.

Beyond glucose and lactate, glutamine represents an important source of TCA cycle carbons. [U-^13^C]glutamine tracing generates M5 glutamate, which is further deaminated or transaminated to fully labeled αKG. Subsequent oxidation of αKG in the TCA cycle produces M4 metabolites by the end of the first round of the cycle (Figure 1H). M5 glutamine was higher in E6.5 compared with E3.5 embryos (Figures 1I and S1C). Accordingly, glutamine contributions to TCA cycle intermediates (M4 glutamate, succinate, aspartate, and citrate) increased in E6.5 embryos compared with E3.5 (Figures 1J, S1C, and S2C). Entering the second round of the TCA cycle, M4 citrate forms M3 αKG and glutamate (Figure 1H). The ratio of M3 (second round) to M5 glutamate (first round) was lower in E6.5 embryos compared with E3.5, suggesting reduced progression of the glutamine-derived carbon in the post-implantation embryos, potentially reflecting differences in TCA oxidative activity, dilution from unlabeled carbon sources, and/or export of TCA intermediates (cataplerosis) (Figures 1K and S2D). Within E6.5 embryos, the EC exhibited a higher M3-to-M5 glutamate ratio than the EPI (Figure S2D). Additionally, succinate dilution (M4 succinate to M5 glutamate) showed a trend of being lower in the ICM than in TE, and in the EC compared with the EPI, which is consistent with the reduced contribution of unlabeled sources in these lineages (Figure S2E), mirroring the lactate labeling experiment. Together, these findings indicate that E6.5 embryos exhibit a more glycolytic metabolism, with likely comparable PDH-dependent entry into the TCA cycle but altered downstream carbon utilization compared with E3.5 embryos. While both glucose and glutamine contribute amply to TCA cycle intermediates, glutamine is the major carbon source in the E6.5 embryos. In addition, differential contributions from nutrients other than glucose or glutamine could account for lineage-specific differences in pre- and post-implantation embryos. Overall, these findings reveal that pre- to post-implantation embryonic metabolism does not undergo a simple switch from a bivalent glycolytic and mitochondrial respiration program to an exclusively glycolytic module. Rather, it is marked by an intricate TCA cycle rewiring, likely enabling key cellular processes to proceed despite the lower oxygen availability in the implanted conceptus.^8^^,^^10^

### TCA rewiring is a shared metabolic hallmark between an embryo’s EPI and EpiLCs

**Figure 2:**
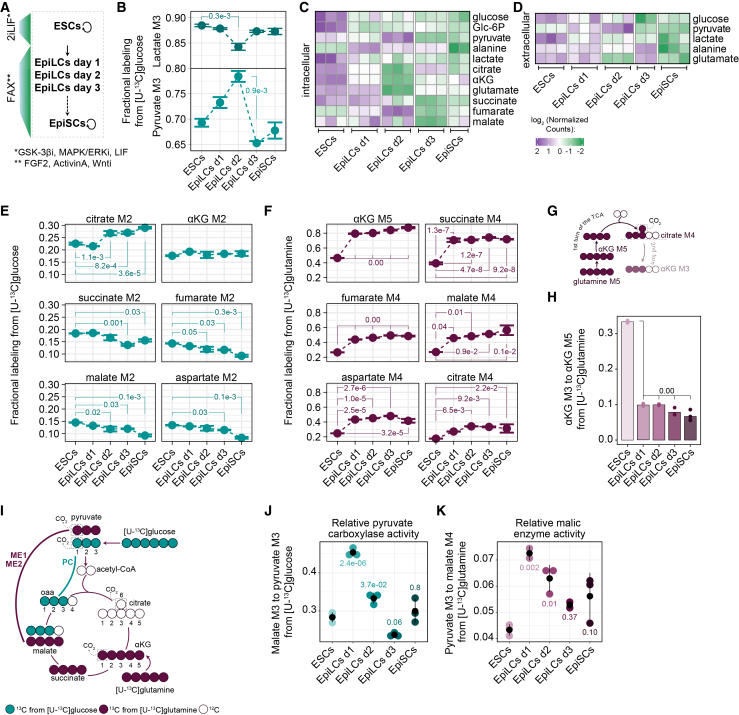
TCA rewiring is a shared metabolic hallmark between embryo’s EPI and EPI-like cells (A) Schematic of the *in vitro* stem cell models. (B) Fractional labeling of M3 pyruvate and lactate from [U-^13^C]glucose. (C and D) Heatmaps of intracellular (C) and extracellular (D) metabolite abundances. Values are ion counts normalized to protein content, scaled across samples and log_2_ transformed. Glc-6P, glucose 6-phosphate; αKG, α-ketoglutarate. (E and F) Fractional labeling of TCA cycle intermediates from [U-^13^C]glucose (E) and [U-^13^C]glutamine (F). (G) Schematic of carbon transitions in two turns of the TCA cycle using a [U-^13^C]glutamine tracer. ^13^C carbons are colored, with the lighter shade indicating a second TCA cycle turn, and unlabeled ^12^C carbons are shown as empty. (H) Ratio of M3 αKG to M5 αKG from [U-^13^C]glutamine. (I) Schematic of carbon transitions following the activities of pyruvate carboxylase (PC) and malic enzymes 1 and 2 (ME1/2). Green and purple indicate ^13^C carbons derived from glucose and glutamine, respectively. Unlabeled ^12^C carbons are shown as empty. (J) Relative PC activity represented by the ratio of M3 malate to M3 pyruvate from [U-^13^C]glucose. (K) Relative ME1/2 activity represented by the ratio of M3 pyruvate to M4 malate from [U-^13^C]glutamine. (B–D, E, F, H, J, and K) Shown are mean values of *n*= 3 except for EpiSCs in (C) and malate M4 in EpiLCs d1 and d2 in (F) where *n* = 2. Plots are ± SEM (B, E, F, J, and K) and/or individual replicate points (H, J, and K). Statistics were calculated with one-way ANOVA with Tukey’s honest significant difference (HSD) test. See also Figures S3 and S4; Table S1.

To refine our understanding of the sequence of metabolic transitions occurring during pre- to post-implantation EPI development in a time-resolved and detailed manner, we employed a stepwise transition from mouse ESCs to EpiLCs and EpiSCs that marks the pluripotency progression (Figure 2A). All cells were cultured in the same basal N2B27 media under 5% O_2_ to mimic the physiological *in vivo* low-oxygen environment. ^13^C nutrients were supplemented for 5 h, by which time isotopic enrichment had reached steady state (Figure S3A), and bulk labeling patterns were analyzed to assess their contribution to glycolytic and TCA cycle intermediates.

Focusing on [U-^13^C]glucose, M3 lactate labeling exceeded 80% throughout the transition, highlighting the highly glycolytic nature of all PSCs (Figure 2B). Although EpiSCs have been described as more glycolytic than ESCs,^17^ M3 lactate remained largely unchanged throughout the transition (Figure 2B). Notably, M3 pyruvate showed a discordance relative to M3 lactate (Figure 2B), remaining approximately 20% lower throughout most of the transition (Figure 2B). This reduction in pyruvate labeling, compared with lactate, suggests the presence of distinct pyruvate pools and/or that unlabeled carbons from alternative metabolic sources and pathways converge on pyruvate.

To better understand the glycolytic changes, we measured intracellular and extracellular levels of glucose, pyruvate, lactate, and other central carbon metabolites (Figures 2C and 2D). Throughout the transition, lactate decreased in both intracellular and extracellular pools, whereas pyruvate fluctuated (Figures 2C and 2D). A rapid decrease in αKG upon EpiLC induction was observed, which is consistent with its function in regulating pluripotency.^18^^,^^20^ Intracellular and extracellular glucose progressively decreased (Figures 2C and 2D), indicating increased consumption during the transition. Despite no significant changes in lactate labeling, the increased glucose uptake suggested enhanced glucose metabolism toward pyruvate and lactate during the transition (Figures S3B and S3C). These data align with the embryo, indicating the post-implantation EPI as more glycolytic. Differences in discrete metabolic nodes, such as lactate labeling, between embryos and PSCs likely reflect divergent nutrient availabilities in both systems.

Next, we investigated how glucose-derived carbons are incorporated into the TCA cycle. M2 citrate increased from EpiLCs day 2 onward (Figures 2E and S3D), suggesting enhanced pyruvate flux through PDH during EpiLC induction. Despite this increased glucose entry into the TCA cycle, M2 αKG remained unchanged and consistently lower than M2 citrate throughout the transition (Figure 2E). Metabolites downstream of αKG, namely succinate, fumarate, and malate, showed a progressive loss in labeling (Figure 2E). This reduced labeling suggests that, at least in part, a significant proportion of citrate could be utilized in pathways other than the TCA cycle. Consistent with this, naive mouse ESCs have been reported to possess a fragmented TCA cycle wherein citrate is exported to the cytoplasm for increased utilization by ACLY.^21^ Our findings further indicate that this feature extends beyond naive to formative and primed pluripotent states too.

Utilizing [U-^13^C]glutamine tracing, we observed significantly higher M5 αKG during the transition, reaching 80% in EpiLCs day 1 compared with 46% in ESCs (Figure 2F). Consistently, the rest of the TCA cycle also exhibited increased labeling (Figure 2F), while the ratio of M3 (second round) to M5 αKG (first round) decreased significantly (Figures 2G and 2H). The latter indicated altered downstream utilization of glutamine-derived carbons, potentially reflecting reduced TCA cycling, increased dilution from alternative sources, and/or enhanced cataplerosis as additional mechanisms. These findings complement previous studies showing that EpiSCs have reduced mitochondrial respiration and require glutamine for proliferation.^17^^,^^18^ Together, our results underscore a fundamental metabolic shift shared between embryos and stem cells, which is characterized by robust glucose/lactate entry into the TCA cycle, enhanced glutamine anaplerosis, and an extensive TCA remodeling in the post-implantation stage of development.

### Pyruvate is a distinctive metabolic node across the pluripotency spectrum

The [U-^13^C]glucose tracing revealed discordant labeling between pyruvate and lactate (Figure 2B), suggesting that carbons stemming from alternative routes converge on pyruvate and further supplement the TCA cycle. Consistently, we observed abundant M3 fumarate, malate, aspartate, and citrate throughout the transition (Figure S3E). While these can arise from the second round of the TCA cycle onward, their fractional enrichment exceeded that of their precursors, namely M4 citrate, M3 αKG, and M3 succinate (Figure S3E), supporting an alternative glucose entry into the TCA cycle independent of PDH. One such route is likely mediated by PC, which converts pyruvate to oxaloacetate (Figure 2I).^37^^,^^38^^,^^39^^,^^40^^,^^41^ This entry significantly spikes in EpiLCs day 1, accounting for up to 33% labeling in fumarate, malate, and aspartate (Figures 2J and S3E–S3H; Table S1). PC-derived carbons eventually flow into citrate, contributing at levels comparable to PDH in EpiLCs day 1 (Figures 2E and S3E–S3H).

In addition to PC, malic enzymes (mitochondrial ME2 and cytoplasmic ME1) interconvert pyruvate to malate (Figure 2I). The [U-^13^C]glutamine tracing revealed labeled pyruvate, alanine, and lactate, with the highest levels in EpiLCs day 1 when normalized to M4 malate (Figures 2K, S3I, and S3J; Table S1). The activity of ME(s) and/or PC was further reflected in the increased M6 citrate during the transition, arising from the condensation of glutamine-derived M2 acetyl-CoA with M4 oxaloacetate (Figure S3K; Table S1). Accordingly, elevated PC and MEs in EpiLCs days 1 and 2 could explain the increased intracellular abundance of fumarate and malate during the formative pluripotency transition (Figure 2C). Collectively, these findings reveal substantial glucose contribution to the TCA cycle through an alternative entry involving PC. This anaplerotic influx appears to be connected to an efflux of intermediates such as malate or citrate. Malate can be reconverted to pyruvate through the MEs, completing a pyruvate-malate cycle; whereas, citrate export followed by ACLY-dependent cleavage and subsequent conversion back to pyruvate forms a pyruvate-citrate cycle. Although the relative activity of these cycles is difficult to estimate, pyruvate cycling is detectable throughout pluripotency, peaking at the onset of formative pluripotency and later equalizing to naive levels (Figures 2J and 2K).

Although many isotopologues indicative of pyruvate cycling were not detected in embryos owing to the limited sensitivity of MALDI-MSI, M3 citrate was present in the [U-^13^C]glucose and [U-^13^C]lactate tracing experiments in both E3.5 and E6.5 embryos (Figures S1A and S1B). M5 glutamate was also detected using the [U-^13^C]lactate tracer, suggesting combined contributions from PC and PDH (Figure S1B). Together, these findings indicate that robust pyruvate re-routing is a defining metabolic feature of stem cells and embryos, positioning pyruvate as a distinctive, developmentally regulated node within central carbon metabolism.

### Pyruvate cycling allows for timely onset of formative pluripotency and further lineage specification

**Figure 3:**
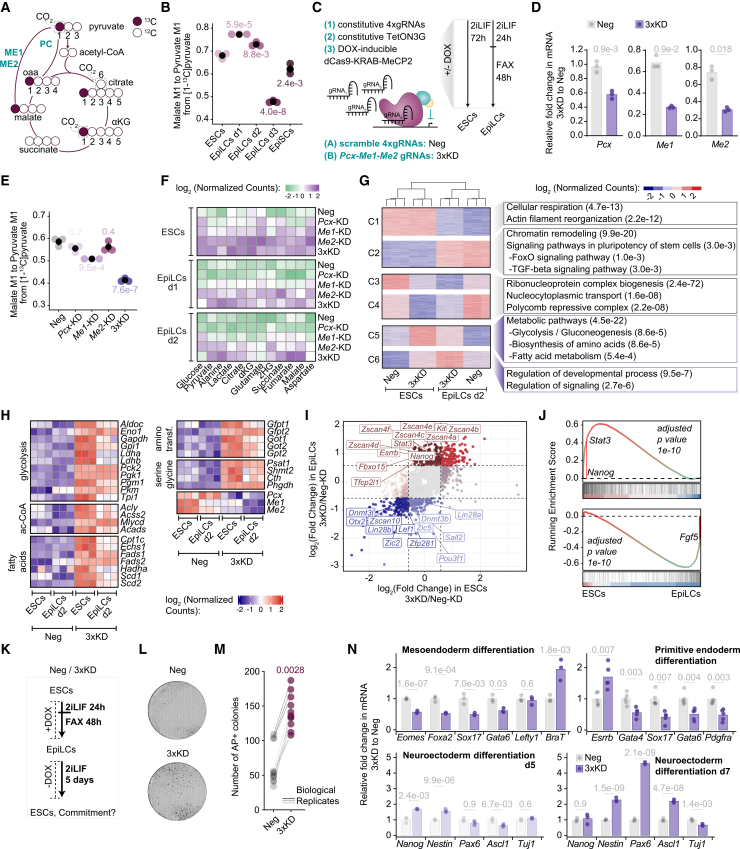
Pyruvate cycling allows for timely onset of formative pluripotency and differentiation (A) Schematic of carbon transitions in the TCA cycle using a [1-^13^C]pyruvate tracer. ^13^C and ^12^C carbons are colored and empty, respectively. (B) Ratio of M1 malate to M1 pyruvate from [1-^13^C]pyruvate. (C) Schematic of the inducible E14-DOX-CRISPRi cell line. The experimental design for doxycycline-induced KD in ESCs and EpiLCs is shown on the right. (D) RT-qPCR analysis of the indicated genes in scramble control (Neg) and simultaneous KD of *Pcx*, *Me1*, and *Me2* (3×KD) in DMSO or DOX conditions. mRNA levels were normalized to *actin* and *Tbp*. (E) Ratio of M1 malate to M1 pyruvate from [1-^13^C]pyruvate in indicated KD lines in EpiLCs day 1. (F) Heatmap of intracellular metabolite abundances upon DOX treatment in indicated KD ESCs and EpiLCs day 1 and day 2. Values are ion counts normalized to protein content, scaled across samples and log_2_ transformed. αKG, α-ketoglutarate; 2HG, 2-hydroxyglutarate. (G) K-means clustering of gene expression data in Neg and 3×KD in ESCs and EpiLCs day 2. The most significant Gene Ontology terms for each cluster are shown. (H) Expression of metabolic genes in 3×KD and Neg ESCs and EpiLCs day 2 treated with DOX. (I) Scatter plot of the log_2_ fold changes in gene expression in 3×KD versus Neg in ESCs and EpiLCs day 2. Specific naive (brown) and primed (blue) pluripotency genes are highlighted. Dashed lines mark a ± 0.58 log_2_ fold change. (J) Gene set enrichment analysis of the significantly upregulated (top) and downregulated (bottom) genes in the 3×KD compared with Neg in EpiLCs day 2. The ranked list was generated from the comparison of Neg ESCs to Neg EpiLCs day 2. (K) Experimental setup for assessing the capacity of EpiLCs to revert to ESCs. (L and M) Representative bright-field images (L) and quantification (M) of alkaline phosphatase-positive (AP^+^) colonies from indicated lines. (N) RT-qPCR analysis showing fold change in expression of the indicated genes in Neg and 3×KD lines following targeted differentiation toward mesoendoderm, primitive endoderm, or neuroectoderm. (B, E, F, I, and N) Shown are mean values of *n* = 3. Plots are ± SEM (B and E) and/or individual replicate points (B, D, E, G, H, and N). (M) Each of 3 technical replicates from *n* = 3 are plotted as points. Statistics were calculated with one-way ANOVA with Tukey’s HSD test (B, D, E, and N) or an unpaired *t* test (M). See also Figures S4 and S5; Table S1. Metabolomic datasets, related to Figures 1, 2, 3, 4, 5, and 6, Table S3. Differential gene expression analysis, related to Figure 3, Document S2. Article plus supplemental information, Table S4. Oligonucleotides and sgRNAs used for this study, related to Figures 3 and 6.

The distinct pyruvate re-routing and dynamics prompted us to further investigate its role in PSCs transitions. First, pyruvate anaplerosis via PC was confirmed using [1-¹³C]pyruvate, which distinguishes PDH- and PC-dependent glucose entry into the TCA cycle. While PDH activity results in loss of ^13^C carbon as ^13^CO_2_, PC-mediated entry retains it in oxaloacetate and downstream TCA intermediates (Figure 3A). Accordingly, the presence of M1 malate, fumarate, aspartate, and citrate confirmed that pyruvate anaplerosis is robust in stem cells and dynamically regulated during EpiLC induction (Figures 3B, S4A, and S4B).

Next, we generated a multiplex acute loss-of-function system targeting PC/ME1/ME2 using a doxycycline (DOX)-inducible CRISPR interference (CRISPRi) ESC line (Figures 3C and S4C–S4J). We established single knockdowns (KDs) for *Pcx*, *Me1*, and *Me2* (Figure S4K) and a triple KD (3×KD) by transfecting a vector harboring guide RNAs (gRNAs) targeting all three genes (Figures 3C, 3D, and S4L–S4N). These ESCs were induced to EpiLCs and were supplemented with [1-^13^C]pyruvate. Repression of *Pcx* and *Me2* alone slightly decreased M1 malate (Figure 3E), whereas *Me1*-KD and particularly the 3×KD significantly caused a marked decrease (Figures 3E and S4O). These results confirmed that pyruvate re-routing is mediated through the concerted actions of PC and ME1/ME2.

Metabolomic profiling of the single KDs and 3×KD in ESCs and through EpiLC induction revealed that each KD exerted similar metabolic alterations, albeit to varying degrees (Figure 3F; Table S1) . Overall, metabolites progressively accumulated, with weakest effects in *Pcx*-KD, intermediate in *Me1*-KD, and strongest in *Me2*-KD and 3×KD. The most consistent alteration in all KDs included increased glucose, glutamate, proline, and aspartate, particularly in ESCs. Focusing on metabolites changing in opposite directions across KDs, pyruvate and lactate increased in all KDs apart from the *Pcx*-KD in which they were significantly reduced. Likewise, citrate increased only following *Me1*, *Me2*, or 3×KD depletion. *Pcx* repression decreased malate, most prominently in EpiLCs day 1, aligning with higher activity at the onset of formative pluripotency, whereas *Me1*, *Me2*, and 3×KD depletion caused malate accumulation. This opposite pattern reflected the substrate-product of the underlying reactions of PC and ME1/ME2, supporting carbon flow from pyruvate to oxaloacetate/malate via PC and back to pyruvate via ME1/ME2, which is consistent with our tracing results. Collectively, these findings highlight the occurrence of pyruvate cycling both in ESCs and EpiLCs. The accumulation of metabolites, particularly upon the combined repression, suggests compensatory adaptations and highlights a regulatory role for pyruvate cycling in bridging TCA flux with stem cell demands for carbon, energy, reducing equivalents, and overall biosynthesis.

To examine the pyruvate cycling impact on cell-state and pluripotency progression, we performed mRNA sequencing on the 3×KD in ESCs and EpiLCs. Unsupervised clustering of differentially expressed genes followed by Gene Ontology (GO) enrichment analysis validated that control EpiLCs downregulated genes involved in cellular respiration (cluster 1) and upregulated genes linked to chromatin remodeling and PSC-related signaling (cluster 2) (Figure 3G). Clusters 3 and 4 represented downregulated genes in both ESCs and EpiLCs upon pyruvate cycling repression, which were enriched for ribonucleoprotein complex biogenesis and regulators of developmental genes. Clusters 5 and 6 included upregulated genes following pyruvate cycling depletion in both ESCs and EpiLCs and were enriched for metabolic terms (Figure 3G). Analysis of central carbon metabolism revealed robust expression of *Pcx*, *Me1*, and *Me2* throughout the developmental transition and efficient repression in the 3×KD (Figure 3H). The pyruvate cycling depletion resulted in upregulation of glycolytic genes and branching pathways, aminotransferases, fatty acid, and acetyl-CoA metabolism (Figure 3H). Notably, key regulators of the pyruvate node and its re-routing, including pyruvate kinase muscle isoform (*Pkm*) and phosphoenolpyruvate carboxykinase 2 (*Pck2*), were among the most upregulated genes, suggesting a coordinated adaptation to enhance glycolytic flux and reinforce alternative pyruvate cycling routes. These transcriptional adaptations mirrored the metabolic phenotype of 3×KD, which is characterized by the accumulation of glucose and glycolytic intermediates (Figure 3F). Consistently, the signaling milieu, comprising pathways that mediate metabolic adaptations, was also deregulated (Figures 3G and S5A). Focusing on genes whose expression is dynamically regulated during the exit from naive pluripotency, 3×KD EpiLCs, but not ESCs, displayed upregulation of naive and downregulation of primed pluripotency markers (Figure 3I). Gene set enrichment analysis (GSEA) further supported this phenotype of delayed exit from naive pluripotency (Figure 3J). Functionally, EpiLC reversion assays (Figure 3K)^42^^,^^43^ confirmed increased reversion efficiency to naive pluripotency in the 3×KD, whereas only a few control cells formed alkaline phosphatase (AP+) colonies (Figures 3L and 3M). Together, these findings indicate that pyruvate cycling is indispensable for timely commitment to formative pluripotency.

Finally, we assessed how depletion of pyruvate cycling influences differentiation (Figure 3N). Control and 3×KD ESCs were differentiated toward mesoendoderm, primitive endoderm, and neuroectoderm in the presence of DOX.^44^^,^^45^^,^^46^ During mesoendoderm differentiation, 3×KD cells showed reduced induction of lineage markers (*Eomes*, *Foxa2*, *Sox17*, and *Gata6*) but elevated *Brachyury* expression (Figure 3N), which is consistent with the antagonism between EOMES and BRACHYURY during early gastrulation.^47^ During primitive endoderm differentiation, loss of pyruvate cycling resulted in decreased expression of endoderm markers (*Gata4*, *Sox17*, *Gata6*, and *Pdgfra*; Figure 3N), but a reciprocal increase in the naive marker *Esrrb*. In neuroectodermal differentiation, 3×KD cells also exhibited elevated expression of the pluripotency marker *Nanog* and the neural progenitor marker *Nestin*, but reduced induction of the later proneural marker *Ascl1* at day 5. By differentiation day 7, *Nestin*, *Pax6*, and *Ascl1* were increased despite reduced expression of the post-mitotic neuronal marker *Tuj1* (Figure 3N). Together, these results indicate that pyruvate cycling supports the proper differentiation progression toward mesendoderm, primitive endoderm, and neuroectoderm.

Collectively, these findings highlight that pyruvate cycling functions as a fundamental metabolic circuit that integrates metabolic homeostasis with developmental timing, thereby influencing both the timely exit from pluripotency and the orderly progression of lineage differentiation programs.

### Reductive carboxylation of αKG fuels citrate production

**Figure 4:**
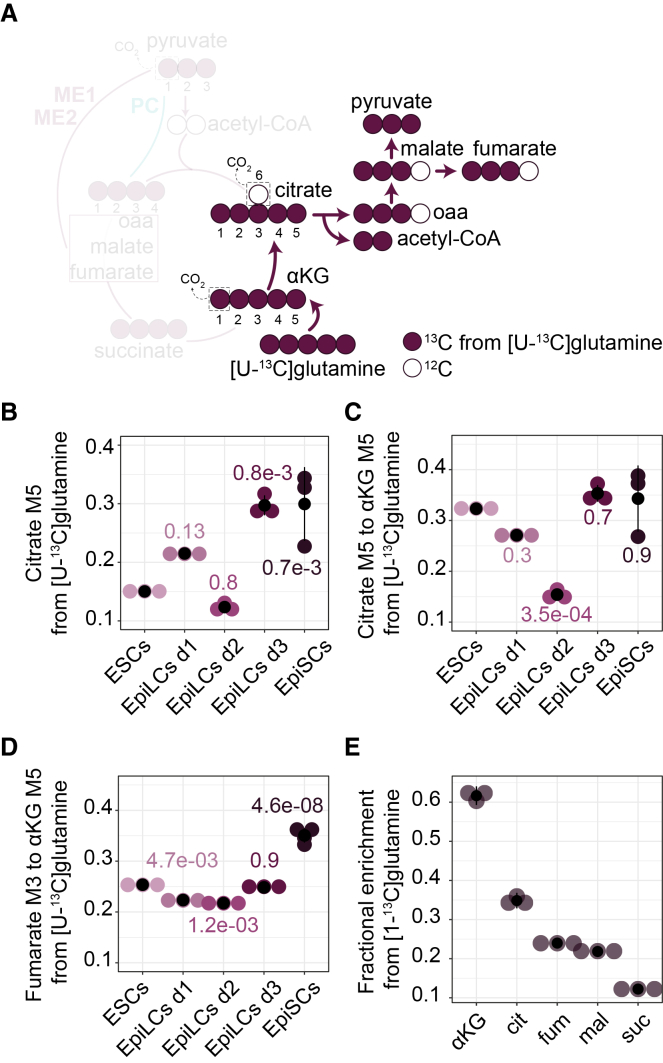
Reductive carboxylation of αKG fuels citrate production (A) Schematic of carbon transitions during reductive glutamine carboxylation with a [U-^13^C]glutamine tracer. ^13^C and ^12^C carbons are colored and empty, respectively. (B) Labeling of M5 citrate from [U-^13^C]glutamine. (C) Relative activity of reductive glutamine carboxylation represented by the ratio of M5 citrate to M5 αKG from [U-^13^C]glutamine. (D) Ratio of M3 fumarate to M5 αKG from [U-^13^C]glutamine. (E) Labeling in M1 isotopologue of the indicated metabolites from [1-^13^C]glutamine in ESCs. (B–E) Shown are mean values of *n* = 3 ± SD with individual replicates plotted as points. (B–D) Statistics were calculated with one-way ANOVA with Tukey’s HSD test. See also Figure S5; Table S1.

Parallel to pyruvate anaplerosis, glutamine contribution to the TCA cycle increased markedly in EpiLCs, EpiSCs, and the E6.5 EPI (Figures 1I, 1J, and 2F). Beyond oxidative metabolism, glutamine can undergo reductive carboxylation, whereby αKG is converted to citrate through the isocitrate dehydrogenase(s) and aconitase(s) (Figure 4A).^48^^,^^49^^,^^50^^,^^51^^,^^52^ [U-^13^C]glutamine tracing revealed the presence of M5 citrate, reaching up to 30% in EpiLCs day 3 and EpiSCs (Figure 4B), indicating a substantial reductive flux. M5 citrate cleaved by ACLY generates M2 acetyl-CoA and M3 oxaloacetate, which subsequently forms M3 malate, fumarate, and aspartate (Figure 4A). Normalized M5 citrate to M5 αKG indicated decreased reductive carboxylation in EpiLCs day 2, whereas normalized M3 malate, fumarate, and aspartate were increased in EpiSCs, suggesting enhanced downstream processing of reductively derived citrate by ACLY and/or subsequent metabolic enzymes (Figures 4C, 4D, and S5B).

[1-^13^C]glutamine tracing in ESCs further validated the occurrence of reductive carboxylation, with M1 isotopologues detected in αKG, citrate, malate, and fumarate, but not in succinate due to loss of the ^13^C as ^13^CO_2_ at the αKG dehydrogenase step (Figure 4E). Together, these data indicated that reductive carboxylation is active throughout pluripotency and linked to ACLY, which generates acetyl-CoA, likely supporting lipid synthesis and acetyltransferase-mediated modifications.

### Glutamine is a more prominent source of carbon for histone acetylation than glucose

**Figure 5:**
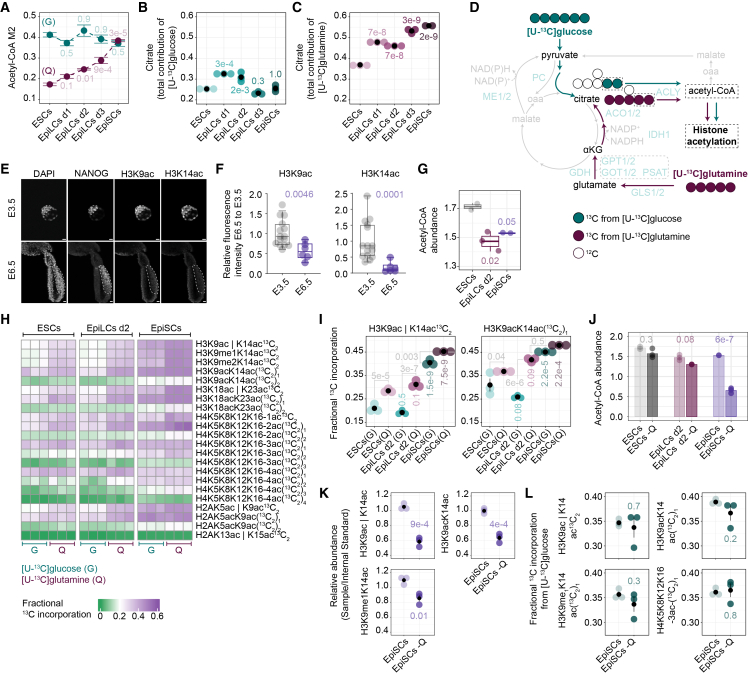
Glutamine is a more prominent source of carbon for histone acetylation than glucose (A) Fractional labeling of M2 acetyl-CoA from [U-^13^C]glucose (G) and [U-^13^C]glutamine (Q). (B and C) Total carbon contribution from [U-^13^C]glucose (B) and [U-^13^C]glutamine (C) to citrate. (D) Schematic of carbon transitions in acetyl-CoA production from citrate using [U-^13^C]glucose or [U-^13^C]glutamine tracing. Green and purple indicate ^13^C carbons derived from glucose and glutamine, respectively. Unlabeled ^12^C carbons are shown as empty. (E and F) Representative whole-mount immunofluorescence images (E) with quantification (F) of NANOG, H3K9ac, and H3K14ac in E3.5 and E6.5 mouse embryos. The dotted line depicts the NANOG-positive region. Visceral endoderm was micro-dissected. Scale bar, 20 μm, *N* > 6. (G) Acetyl-CoA abundance levels. Values are ion counts normalized to protein content. (H and I) Plots showing the fractional ^13^C labeling of indicated histone acetylated peptides from [U-^13^C]glucose (G) and [U-^13^C] glutamine (Q). (J) Acetyl-CoA abundance levels in glutamine-replete and glutamine-deprived (−Q) conditions. Values are ion counts normalized to protein content. (K) Relative levels of specific histone acetylation marks (sample versus internal standard) in EpiSCs in glutamine-replete and deprived (−Q) conditions. (L) Fractional ^13^C labeling of indicated histone acetylated peptides from [U-^13^C]glucose in EpiSCs in glutamine-replete and deprived (−Q) conditions. (H, I, and L) Residues separated by “l” indicate the presence of the modification on one residue or the other. (A–C and G–L) Shown are mean values of *n* = 3 except for EpiLCs d2/3 in (A), ESC/EpiSC in (G), ESCs/EpiLCs d2 −Q/EpiSCs in (J), where *n* = 2. Plots are ± SEM (A–C, I, K, and L), and/or individual replicate points (A–C, F, G, and I–L), and/or boxplots with the median, the interquartile range, and whiskers indicating the minimum and maximum values (F and G). Statistics were calculated with one-way ANOVA (A–C and J–L) or two-way ANOVA (I) with Tukey’s HSD test or a Mann-Whitney unpaired test (F). See also Figure S5; Table S1.

Owing to the increased contribution of glutamine to TCA cycle intermediates in the formative and primed pluripotent states (Figures 1I, 1J, and 2F), and the robust activity of reductive carboxylation throughout pluripotency (Figures 4B–4E), we hypothesized that glutamine, through citrate synthesis, may contribute substantially to acetyl-CoA pools utilized for histone acetylation. Although glucose-derived acetyl-CoA has traditionally been considered the primary source of histone acetylation,^53^^,^^54^ we sought to determine whether this paradigm holds across the pluripotency spectrum. Therefore, we quantified M2 acetyl-CoA following [U-^13^C]glucose or [U-^13^C]glutamine tracing. Glucose robustly contributed to acetyl-CoA synthesis across all pluripotent states (Figure 5A), as previously reported.^53^^,^^54^^,^^55^ In contrast, glutamine-derived acetyl-CoA increased progressively as cells transitioned out of naive pluripotency, ultimately reaching levels comparable to glucose in EpiSCs (Figure 5A). Consistently, total glutamine contribution to citrate exceeded that of glucose already in naive cells and peaked in primed pluripotency (Figures 5B and 5C). Together, these findings suggest the existence of a compartmentalized glutamine-derived acetyl-CoA pool used for histone acetylation.

To elucidate the main metabolic source supporting acetylation directly at the histone level, we supplemented PSCs with [U-^13^C]glucose or [U-^13^C]glutamine for 5 h, when labeling reaches a steady state, and quantified the ^13^C incorporation in acetylated histone peptides by mass spectrometry (Figures 5D and S5C). Focusing first on the relative abundance of individual histone modifications, we confirmed a robust increase in H3K9me2 and a concomitant loss of H3K27me3 at the onset of formative pluripotency (Figure S5D).^23^ Global levels of histone acetylation marks were dynamic, with H3K9ac and H3K14ac decreasing in primed compared with naive pluripotency, as is also the case in the embryo (Figures 5E, 5F, S5G, and S5H). Consistent with this hypoacetylation state, the acetyl-CoA levels were significantly lower in the formative and primed PSCs compared with the naive (Figure 5G).

Next, to define the dominant carbon source for histone acetylation, we analyzed the ^13^C incorporation in acetylated histone peptides. Across all pluripotent states, glutamine-derived carbons contributed more to histone acetylation than glucose (Figures 5H, 5I, S5E, and S5F). Moreover, this ^13^C incorporation progressively increased from the naive to the formative and primed pluripotent states (Figures 5H and 5I). Collectively, these results revealed glutamine as the predominant carbon source fueling the histone acetylation backbone throughout pluripotency.

To functionally test whether glutamine sustains acetyl-CoA and histone acetylation, we deprived naive, formative, and primed PSCs of glutamine for 5 h and quantified the abundance of acetyl-CoA. Glutamine depletion markedly reduced acetyl-CoA levels in EpiSCs, while ESCs and EpiLCs showed a modest, non-significant downward trend (Figure 5J). To assess the impact on histone acetylation marks, histone post-translational modifications (PTMs) profiling was performed in glutamine-replete and -deprived EpiSCs, revealing a significant reduction in H3K9ac and H3K14ac marks upon glutamine withdrawal (Figure 5K). To assess whether glucose compensates for glutamine loss by sustaining acetyl-CoA biosynthesis, EpiSCs were supplemented with [U-^13^C]glucose under glutamine-deprived conditions. Glucose contribution to acetyl-CoA did not increase (Figure S6A), and this lack of compensation was reflected in the histone ^13^C labeling (Figures 5L and S6B), indicating limited metabolic plasticity and suggesting compartmentalized production and preferential routing of glutamine-derived acetyl-CoA to chromatin acetylation. Collectively, our data highlight that glutamine is not merely an auxiliary carbon source; rather, it constitutes the major metabolic source for acetyl-CoA biosynthesis routed toward histone acetylation.

### Glutamine fuels histone acetylation through the IDH1-mediated reductive carboxylation

**Figure 6:**
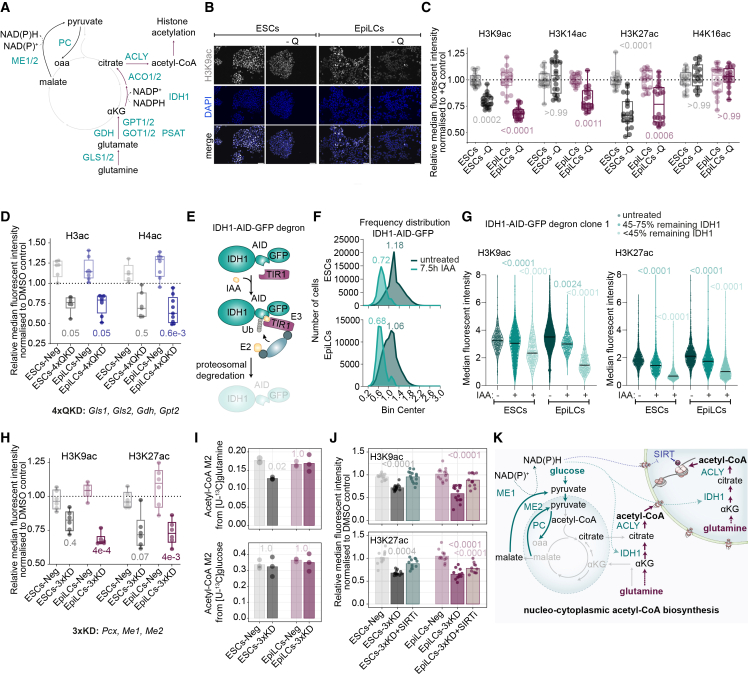
Reductive glutamine carboxylation and pyruvate cycling cooperate to sustain histone acetylation levels (A) Schematic of selected reactions in the TCA cycle and auxiliary pathways with key enzymes. (B and C) Representative immunofluorescence images (B) with quantification (C) of histone acetylation levels in ESCs and EpiLCs day 2 in glutamine-replete and glutamine-deprived conditions. Scale bar, 50 μm. (D) Relative median fluorescent intensity of indicated histone acetylation marks in Neg and 4×QKD cell lines in ESCs and EpiLCs day 2. (E) Schematic representation of the IDH1-AID-GFP degron cell line. IAA, indole-3-acetic acid. (F) Frequency distribution of nuclear mean GFP intensity in untreated and IAA-treated (7.5 h) IDH1-AID-GFP ESCs and EpiLCs day 2. (G) Median fluorescence intensity of the indicated histone acetylation marks in IDH1-AID-GFP ESCs and EpiLCs (day 2). Cells were stratified according to residual IDH1-AID-GFP levels as visualized in (F). (H) Relative median fluorescent intensity of indicated histone acetylation marks in Neg and 3×KD cell lines in ESCs and EpiLCs day 2. (I) Fractional labeling of M2 acetyl-CoA from [U-^13^C]glutamine (upper) and [U-^13^C]glucose (lower) in Neg and 3×KD ESCs and EpiLCs day 2. (J) Relative median fluorescent intensity of indicated histone acetylation marks in Neg and 3×KD cell lines in ESCs and EpiLCs day 2 with or without sirtuin inhibition for 24 h (SIRTi: NAM). (K) A proposed model in which the TCA cycle becomes rewired during development under low oxygen levels. (C, D, and G–J) Shown are values from *n* = 6 (H), *n* = 5 (C, D, G, and J), and *n* = 3 (I), except for ESCs-Neg (upper)/EpiLC-Neg (lower) in (I), where *n* = 2. Plotted is the mean (I and J), median (G), and/or individual replicate points (C, D, G, and H–J), and/or boxplots with the median, the interquartile range, and whiskers indicating the minimum and maximum values (C, D, and H). Each biological replicate includes 4 technical replicates (C) with at least 20,000 (C, D, H, and J) or 800 (G) cells quantified. Statistics were calculated using the Kruskal-Wallis test with Dunn’s post hoc test, with and Benjamini-Hochberg adjustment of *p* values (C, D, G, and H) or one-way ANOVA followed by Tukey’s HSD post hoc test (I) or two-way ANOVA followed by Sidák’s multiple comparisons test (J). See also Figure S6; Tables S1 and S4.

Given the essential contribution of glutamine to histone acetylation, we sought to examine the key enzymatic steps that channel glutamine-derived carbons toward acetyl-CoA using our E14-CRISPRi-DOX line in ESCs and EpiLCs (Figures 6A and S4C–S4J). First, we confirmed that glutamine-deprived ESCs and EpiLCs showed reduced levels of histone acetylation (Figures 6B and 6C). Next, two CRISPRi-based quadruple KD cell lines were generated: one targeting the initial steps of glutamine processing (*Gls1*, *Gls2*, *Gdh*, and *Gpt1*; 4×QKD), and a second targeting aminotransferases (*Got1*, *Got2*, *Psat*, and *Gpt1*; 4×TKD) (Figures 6A, S6C, and S6D). While repression of aminotransferases did not significantly affect histone acetylation marks (Figure S6E), KD of the early glutamine-processing enzymes markedly reduced global H3 and H4 acetylation in both ESCs and EpiLCs (Figure 6D), phenocopying glutamine deprivation (Figures 6B and 6C).

Based on our tracing data, we hypothesized that acetyl-CoA supply to chromatin proceeds predominantly through the reductive carboxylation mediated by the cytoplasmic and nuclear IDH1, which catalyzes the carboxylation of αKG to isocitrate (Figure 6A).^3^^,^^49^ To test this, we generated an auxin-inducible IDH1 degron ESC line (Figures 6E and S6F–S6H). Degron ESCs and EpiLCs were treated with indoleacetic acid (IAA) for 7.5 h, leading to a 30% reduction in the median nuclear frequency distribution of IDH1-AID-GFP, without compromising cell viability (Figure 6F). Due to the heterogeneous response to IAA under these acute conditions, cells were stratified by residual IDH1 abundance, which revealed a dose-dependent reduction in H3K9ac and H3K27ac upon IDH1 loss (Figures 6G and S6I). Collectively, these findings uncover that glutamine, through IDH1-mediated reductive carboxylation, serves as a major source of acetyl-CoA for histone acetylation in PSCs.

### Pyruvate cycling and reductive glutamine carboxylation cooperate to sustain histone acetylation

Reductive carboxylation is sensitive to the cellular NAD(P)+/NAD(P)H ratio.^49^^,^^56^^,^^57^ Because pyruvate cycling also generates redox cofactors through the activity of MEs (Figure 6A), we hypothesized that these two pathways could be metabolically coupled and together regulate the glutamine-dependent acetyl-CoA pool used for histone acetylation. Depletion of pyruvate cycling (3×KD) resulted in H3K9ac and H3K27ac decrease, resembling the effects of glutamine withdrawal (Figures 6H and S6J). We next assessed whether this phenotype reflected impaired acetyl-CoA availability. Although total acetyl-CoA levels were unchanged (Figure S6K), isotope tracing revealed a significant but modest decrease in M2 acetyl-CoA selectively from [U-¹³C]glutamine in ESCs (Figures 6I and S6L). This selective reduction indicated that pyruvate cycling does indeed support the glutamine-derived acetyl-CoA pool, which is consistent with a role for NADPH-dependent reductive carboxylation mediated by IDH1 in supplying acetyl-CoA for histone acetylation. In contrast, EpiLCs showed minimal changes, suggesting a cell-type-specific magnitude and dynamics of coupling between pyruvate cycling and reductive carboxylation and/or that additional mechanisms are at play.

To further investigate the histone hypoacetylation phenotype, we utilized the sirtuin (SIRT) inhibitor nicotinamide (NAM).^58^ Because SIRTs are NAD^+^ dependent, this allowed us to functionally separate the NADPH-dependent acetyl-CoA supply component and interrogate possible increased deacetylase activity. NAM treatment rescued the reduction in histone acetylation in pyruvate cycling-deficient cells, restoring acetylation levels to near baseline, especially of the SIRT-sensitive H3K9ac (Figure 6J).^59^ These findings demonstrated that histone hypoacetylation upon pyruvate cycling depletion did not result from a reduced bulk acetyl-CoA level, but instead likely reflected increased SIRT-dependent deacetylation acting in parallel with altered acetyl-CoA sourcing.

More broadly, these data indicate that histone acetylation is governed not simply by total acetyl-CoA abundance but by nutrient- and route-specific generated acetyl-CoA pools (Figure 6K). In this context, pyruvate cycling and IDH1-mediated reductive carboxylation are emerging as tightly interconnected metabolic nodes central to coupling glutamine metabolism to histone acetylation.

## Discussion

Our study reveals that central carbon metabolism undergoes dynamic rewiring at the time of implantation. While previous studies suggested a simple metabolic switch from a bimodal glycolytic-oxidative program in naive cells to an exclusively glycolytic in primed cells,^9^^,^^17^ we uncover that implantation is characterized by a spatio-temporal rewiring of the TCA cycle. The substantial glucose-derived carbon entry into the TCA cycle that we observe is consistent with glucose contributing to TCA intermediates only from the blastocyst stage onward.^6^ Rather than an overall shutdown of the TCA cycle, implantation involves robust glucose entry through PDH, likely supporting biosynthetic demands, as citrate can be exported to the cytosol and cleaved by ACLY to produce acetyl-CoA.^54^ Indeed, *Acly-*KO ESCs fail to exit naive pluripotency upon 2iLIF withdrawal.^21^ This suggests that the heightened proliferation and low-oxygen conditions at implantation likely increase the demand for acetyl-CoA to support lipid biosynthesis and protein acetylation.

In parallel, we uncover substantial PC-mediated TCA cycle anaplerosis across pre- and post-implantation embryos and pluripotent states, which is consistent with detectable, albeit lower, PC activity in ESCs and blastocysts cultured under atmospheric oxygen levels.^6^^,^^19^ The importance of PC has been mainly described beyond embryonic contexts. In the liver, PC is essential for TCA cycle replenishment, gluconeogenesis, and redox homeostasis.^37^ In certain tumors, PC functions as the primary anaplerotic route enabling glutamine-independent growth, with its deletion compromising tumor formation.^36^^,^^38^^,^^39^^,^^40^^,^^41^ Broadly, PC supports biosynthesis and thus metabolic flexibility, processes that, as our findings suggest, are also critical for pluripotent state transitions.

Notably, PC-mediated anaplerosis is accompanied by reverse flux through MEs that regenerate pyruvate, forming a pyruvate cycling pathway similar to that described in differentiating adipocytes, where it supports NADPH and cytosolic acetyl-CoA biosynthesis.^60^^,^^61^ Stem cells lacking pyruvate cycling show altered differentiation dynamics. The reasons for this could be manifold, including the accumulation of αKG upon *Pcx/Me1/Me2* KD, a metabolite known to sustain naive pluripotency^18^^,^^20^; as well as broader effects on the signaling milieu, which could stabilize naivety; and on the redox and biosynthetic states that support differentiation. As differential glucose metabolism regulates gastrulation through the hexosamine biosynthetic pathway rather than the final pyruvate kinase M2 (PKM2)-dependent glycolytic step,^11^ our finding that the post-implantation EPI fuels the TCA cycle from exogenous lactate, thereby bypassing PKM2, raises the possibility that pyruvate metabolism may play a broader developmental role, including during gastrulation.

Beyond pyruvate, glutamine anaplerosis rises in formative and primed EPI both *in vivo* and *in vitro*, aligning with the requirement of Serum/LIF ESCs and EpiSCs for glutamine to proliferate.^18^^,^^19^ Importantly, we identify substantial glutamine reductive carboxylation across pluripotency, generating citrate and acetyl-CoA. This pathway is typically active in highly proliferative systems such as cancers, where it supports lipid biosynthesis, particularly under hypoxia or impaired mitochondrial respiration.^49^^,^^50^^,^^51^^,^^52^ Although low levels of reductive glutamine metabolism have been observed in normoxic PSCs,^62^^,^^63^ our data demonstrate that under physiological oxygen conditions this pathway is manyfold more prominent, positioning glutamine not only as an anaplerotic substrate but also as a major source of citrate and downstream acetyl-CoA. Although acetyl-CoA can originate from pyruvate, citrate, acetate, or lipids,^64^ glucose-derived citrate is typically the major precursor for histone acetylation,^53^^,^^54^^,^^55^ while acetate contributes minimally.^53^^,^^55^ In contrast, we find that across mouse pluripotency, glutamine constitutes the predominant carbon source for histone acetylation, which is consistent with reports in other systems where impaired glutamine metabolism reduces histone acetylation.^65^ Mechanistically, this route involves IDH1-mediated reductive carboxylation and is metabolically coupled to pyruvate cycling, which sustains the redox environment required by IDH1 (Figure 6K). In this way, pyruvate cycling and reductive glutamine metabolism are functionally linked to the generation of acetyl-CoA that fuels histone acetylation. Given the cytoplasmic and nuclear localization of IDH1,^3^^,^^49^ these findings support a model in which chromatin acetylation in PSCs depends on nutrient- and route-specific acetyl-CoA, rather than simply on bulk acetyl-CoA abundance.

In parallel, we identify an unexpected increase in histone acetylation labeling upon exit from naive pluripotency.^66^ This likely reflects increased histone replacement during cell-cycle shortening^67^ and enhanced deacetylase activity as histone acetylation is repositioned during the ESC-to-EpiLC transition.^25^^,^^68^ Functionally, reduced histone acetylation upon loss of pyruvate cycling impairs exit from naive pluripotency, aligning with the requirement for fast acetylation-deacetylation dynamics during development and lineage priming.^26^^,^^27^^,^^28^^,^^29^^,^^30^^,^^31^^,^^69^^,^^70^^,^^71^

Overall, our study uncovers a remarkable complexity in how central carbon metabolism is reorganized at implantation, revealing route-specific nutrient utilization strategies that directly connect metabolic flux to epigenome and pluripotency state transitions. This metabolic architecture appears essential for maintaining developmental progression and enables rapid epigenetic remodeling during early embryogenesis.

### Limitations of the study

This study has several limitations. First, *ex vivo* embryo culture combined with ^13^C tracing may induce metabolic adaptations. Second, MALDI-MSI has limited sensitivity and mass resolution, preventing confident identification of all isotopologues and introducing technical variability that can obscure lineage-specific metabolic states. Quantifying acetyl-CoA in embryos is also constrained by minimal sample availability. Third, *in vitro* PSCs do not fully recapitulate embryonic metabolism and can acquire culture-specific adaptations. Fourth, although our data suggest coupling between pyruvate cycling and reductive glutamine carboxylation, future studies are needed to directly define redox state changes across developmental transitions and the contribution of pyruvate cycling to redox control. Finally, determining how much glucose-derived acetyl-CoA can support histone acetylation will be important for defining the limits of metabolic compensation in pluripotent cells.

## Resource availability

### Lead contact

Further information and requests for resources and reagents should be directed to and will be fulfilled by the lead contact, Jan Żylicz (jan.zylicz@sund.ku.dk).

### Materials availability

All unique reagents generated in this study are available from the lead contact with a completed materials transfer agreement.

### Data and code availability

Sequencing data have been deposited at the Gene Expression Omnibus (GEO) as GSE: 294457 and are publicly available as of the date of publication.

This paper does not report any original code.

Any additional information required to reanalyze the data reported in this paper is available from the lead contact upon request.

## Acknowledgments

This work was supported by grants from Novo Nordisk Fonden (NNF23SA0087869, NNF21CC0073729 and NNF23SA0084103), Lundbeckfonden (R345-2020-1497 and R380-2021-1519), the European Research Council (101077271), European Union (MSCA-PF, 101209861) and Danmarks Frie Forskningsfond (0169-00031B). We thank the Heard lab for the TIR1-ARF16 construct; Robert Bone for feedback; and Alessandro Ghiringhelli, Viktoria Lavro, and Anne Wenzel for experimental or computational support. We thank reNEW and CPR platforms for technical expertise, support, and use of equipment. We particularly wish to thank H. Wollmann, M. Michaut, J. Bulkescher, A. Georgantzoglou, G. Dela Cruz, and A. Kalvisa.

## Author contributions

Conceptualization, E.K., D.P.-M., and J.J.Ż.; methodology, E.K., D.P.-M., G.W., R.N., T.B., A.C.-M., T.J.R., T.M., and J.J.Ż.; investigation, E.K., D.P.-M., L.A.-M., G.W., R.N., S.B.-A., A.C.-M., J.G., M.A.-M., R.S.-A., and J.J.Ż.; formal analysis, E.K., D.P.-M., L.A.-M., G.W., R.N., M.A.-M., T.M., and J.J.Ż.; data curation, E.K., D.P.-M., G.W., R.N., and T.M.; visualization, E.K., D.P.-M., G.W., R.N., and J.J.Ż.; writing—original draft preparation, E.K., D.P.-M., and J.J.Ż.; writing—review and editing, E.K., D.P.-M., L.A.-M., G.W., R.N., R.S.-A., T.B., T.J.R., T.M., and J.J.Ż.; funding acquisition and supervision, E.K., T.B., T.J.R., T.M., and J.J.Ż.; resources, E.K., T.B., T.J.R., and T.M.; validation, E.K., D.P.-M., L.A.-M., G.W., R.N., S.B.-A., J.G., M.A.-M., and R.S.-A.; project administration, E.K. and J.J.Ż.

## Declaration of interests

The authors have no conflict of interest to declare.

## Declaration of generative AI and AI-assisted technologies in the writing process

During the preparation of this work, the authors used Copilot in order to proofread the manuscript. After using this tool or service, the authors reviewed and edited the content as needed and take full responsibility for the content of the publication.

## STAR★Methods

### Key resources table

**Table:** 

REAGENT or RESOURCE	SOURCE	IDENTIFIER
**Antibodies**		

mouse monoclonal anti-H3K9ac (clone MABI0305)	GeneTex	Cat# GTX50898; RRID: AB_2887765
rabbit monoclonal anti-H3K14ac (clone EP964Y)	abcam	Cat# ab52946; RRID: AB_880442
mouse monoclonal anti-H3K27ac (clone MABI0309)	Active Motif	Cat# 39685; RRID: AB_2793305
mouse monoclonal anti-H4K16ac	Diagenode	Cat# C15200219; RRID: AB_3083767
rabbit polyclonal anti-histone H3ac (pan-acetyl)	Active Motif	Cat# 61937;RRID: AB_2793714
rabbit polyclonal anti-histone H4ac (pan-acetyl)	Active Motif	Cat# 39026;RRID: AB_2687872
mouse monoclonal anti-H3K9me2 (clone mAbcam 1220)	abcam	Cat# ab1220;RRID: AB_449854
mouse monoclonal anti-IDH1 (clone GT1521)	ThermoFisher Scientific	Cat# MA5-27759; RRID: AB_2735231
Mouse monoclonal anti-Vinculin	Merck-Sigma-Aldrich	Cat# V9264; RRID: AB_10603627
rat monoclonal anti-Nanog (clone eBioMLC-51)	ThermoFisher Scientific	Cat# 14-5761-80; RRID: AB_763613
mouse monoclonal anti-Cas9	Cell Signaling	Cat# 14697; RRID: AB_2750916
Rabbit polyclonal anti-Lamin B1	abcam	Cat# ab16048; RRID: AB_443298
mouse monoclonal anti-PC (clone 3H2AD9)	abcam	Cat# 110314; RRID: AB_10861166
rabbit monoclonal anti-ME1 (clone EPR28891-30)	abcam	Cat# ab322401; RRID: AB_3698262
rabbit monoclonal anti-ME2 (clone EP7217)	abcam	Cat# ab139686; RRID: AB_3676026
mouse monoclonal anti-beta actin (clone 2D4H5)	proteintech	Cat# 66009-1-Ig; RRID: AB_2687938
goat anti-rabbit IgG H&L (HRP)	abcam	Cat# ab205718; RRID: AB_2819160
goat anti-mouse igG H&L (HRP)	abcam	Cat# ab205719; RRID: AB_2755049
goat anti-mouse (H&L) Alexa Fluor Plus 647	ThermoFisher Scientific	Cat# A32728, RRID: AB_2633277
donkey anti-rabbit (H&L) Alexa Fluor Plus 647	ThermoFisher Scientific	Cat# A32795, RRID: AB_2762835
goat anti-mouse (H&L) Alexa Fluor Plus 488	ThermoFisher Scientific	Cat# A32723, RRID: AB_2633275
goat anti-rabbit (H&L) Alexa Fluor Plus 568	ThermoFisher Scientific	Cat#A11036, RRID: AB_10563566
goat anti-rat (H&L) Alexa Fluor Plus 647	ThermoFisher Scientific	Cat#A48265TR, RRID: AB_2896334

**Chemicals, peptides, and recombinant proteins**		

Leukemia inhibitory factor (LIF)	Produced in house	N/A
PD0325901 (Mek inhibitor)	Axon Medchem	Cat# 1408
CT99021 (GSKb inhibitor)	Axon Medchem	Cat# 1386
Fgf2	Qkine	Cat# Qk025
Activin A	Qkine	Cat# Qk005
XAV939 (Wnt inhibitor)	Merck Sigma-Aldrich	Cat# X3004
DMEM/F12, GlutaMAX	Gibco	Cat# 10565018
Advanced DMEM/F12	Gibco	Cat# 12634010
RPMI 1640, GlutaMAX	Gibco	Cat# 61870036
Neurobasal	Gibco	Cat# 21103-049
B-27 supplement (50x)	Gibco	Cat# 17504044
B-27 supplement (50x), minus insulin	Gibco	Cat# A1895601
N-2 supplement (100x)	Gibco	Cat# 17502048
Dimethyl sulfoxide (DMSO)	Merck Sigma-Aldrich	Cat# D8418
Doxycycline (DOX)	Merck Sigma-Aldrich	Cat# D9891
16% formaldehyde	ThermoFisher Scientific	Cat# 28908
[U-^13^C]lactate	Merck Sigma-Aldrich	Cat# 485926
[U-^13^C]glucose	Merck Sigma-Aldrich	Cat# 389374
[U-^13^C]glutamine	Cambridge Isotope Laboratories, Inc.	Cat# CLM-1822-H-0.1MG
[1-^13^C]sodium pyruvate	Merck Sigma-Aldrich	Cat# 490709
[1-^13^C]glutamine	Cambridge Isotope Laboratories, Inc.	Cat# CLM-3612-1
DMEM/F12 no glucose, no glutamine, no glutamate, no sodium pyruvate	Sartorius-In vitro	Custom-made, Cat# A2477501
Neurobasal, no glucose, no sodium pyruvate	Gibco	Cat# A2477501
b-mercaptoethanol	Merck Sigma-Aldrich	Cat# M3148
Sodium pyruvate (100mM)	Gibco	Cat# 11360070
Non-essential AA (100x)	Gibco	Cat# 11140035
GlutaMAX (100x)	Gibco	Cat# 35050061
rhLaminin-521	Gibco	Cat# A29249
Accutase	StemPro	Cat# A1110501
basticidin	Merck Sigma-Aldrich	Cat# SBR00022
puromycin	Merck Sigma-Aldrich	Cat# P8833
hygromycin B	ThermoFisher Scientific	Cat# 10687010
Janelia Fluor®646 HaloTag® Ligand	Promega	Cat# GA1120
Acetonitrile	Biosolve	Cat# 01204102
Formic acid 0.1%	Biosolve	Cat# 23244102
LC-MS H_2_0	Biosolve	Cat# 23214102
3-nitrophenylhydrazine (3NPH)	Merck Sigma-Aldrich	Cat# N21804
N-(3-dimethylaminoproply)-N’-ethylcarboiimide hydrochloride (EDC)	Merck Sigma-Aldrich	Cat# E7750
2,6-Di-tert-butyl-4-methylphenol (BHT)	Merck Sigma-Aldrich	Cat# B1378
Methoxamine (MOX) reagent	Merck Sigma-Aldrich	Cat# TS-45950
Bis(trimethylsilyl)trifluoroacetamide (BSTFA) with 1% trimethylchlorosilane (TMCS)	ThermoFisher Scientific	Cat# TS-38831
Nicotinamide	Merck Sigma-Aldrich	Cat# N0636

**Critical commercial assays**		

P3 primary cell 4D-nucelofector X	Lonza	Cat# LZG-V4XP-3012
Qiagen RNeasyMini Kit	Qiagen	Cat# 74104
SuperScript™ III First-Strand Synthesis System	ThermoFisher Scientific	Cat# 18080051
NEBNext® Ultra II directional RNA library prep kit for Illumina®	New England Biolabs	Cat# E7765
NEBNext rRNA Depletion Kit v2	New England Biolabs	Cat# E7400S
NEBNext Multiplex Oligos for Illumina Dual Index Primers Set 2	New England Biolabs	Cat# E7780S
Qubit dsDNA HS Assay Kit	ThermoFisher Scientific	Cat# Q33231
Qubit RNA HS Assay Kit	ThermoFisher Scientific	Cat# Q32852

**Deposited data**		

RNA-seq datasets	This study	GSE294457

**Experimental models: Cell lines**		

Mouse: ESC E14J	Brickman Lab	N/A
Mouse: EpiSC E14J	Zylicz Lab	This study
Mouse: ESC E14-DOX-CRISPRi	Zylicz Lab	This study
Mouse: ESC E14-DOX-CRISPRi Neg	Zylicz Lab	This study
Mouse: ESC E14-DOX-CRISPRi Pcx-KD	Zylicz Lab	This study
Mouse: ESC E14-DOX-CRISPRi Me1-KD	Zylicz Lab	This study
Mouse: ESC E14-DOX-CRISPRi Me2-KD	Zylicz Lab	This study
Mouse: ESC E14-DOX-CRISPRi Pcx-Me1-Me2 3xKD	Zylicz Lab	This study
Mouse: ESC E14-DOX-CRISPRi Gls1-Gls2-Gdh-Gpt2 4xQKD	Zylicz Lab	This study
Mouse: ESC E14-DOX-CRISPRi Got1-Got2-Psat-Gpt1 4xTKD	Zylicz Lab	This sutdy
Mouse: ESC E14 TIR1-ARF16; IDH1-AID-GFP	Zylicz Lab	This study

**Experimental models: Organisms/strains**		

Mouse: Swiss	Janvier Labs	Rj:SWISS

**Oligonucleotides**		

RT-qPCR primers	This study	See Table S4
sgRNAs	This study	See Table S4

**Recombinant DNA**		

pCAG-TetON3G	This study	N/A
pTetO-dCas9-KRAB-MeCP2-HaloTag	This study	N/A
pSpCas9(BB)-2A-Puro (PX459) V2.0	Ran et al.^72^	Addgene plasmid#62988
PiggyBac-1xsgRNA	This study	N/A
PiggyBac-4xsgRNA	This study	N/A
pCAG-TIR1-ARF16	Heard Lab	N/A
Idh1-aid-gfp targeting plasmid	This study	N/A
pBaso	Wang et al.^73^	Wang et al.^73^

**Software and algorithms**		

Fiji (ImageJ) 1.54f	Schindelin et al.^74^	RRID: SCR_002285
ScanR analysis (version 3.2.0, r4066 x64)	Olympus	N/A
Spotfire	TIBCO®	RRID: SCR_008858
FCS Express 7 software	De Novo Software	RRID: SCR_016431
Prism v10.2.2	GraphPad	RRID: SCR_002798
Harmony high-content imaging and analysis software	Perkin – Elmer	RRID: SCR_023543
R v4.3.2	R Core Team	RRID: SCR_001905

**Other**		

LightCycler 480	Roche	RRID: SCR_018626
IXplore Spinning Disk Confocal	Olympus	RRID: SCR_021100
Confocal SP8	Leica	RRID: SCR_018169
Cytek® Aurora Spectral Flow Cytometer	Cytek	N/A
A400 TapeStation	Agilent	N/A
NextSeq 2000	Illumina	RRID: SCR_023614
Qubit Fluorometer	Invitrogen	RRID: SCR_026883
Opera Phenix high content spinning disk confocal	Perkin – Elmer	RRID: SCR_021100
ACQUITY HSS T3, 100×2.1 mm, 1.7 μm	Waters	Cat# 186009468

### Experimental model and study participant details

All mouse embryo work was carried out in accordance with European legislation, authorized by the Danish National Animal Experiments Inspectorate (Dyreforsøgstilsynet, license no. 2020-15-0201-00609 and 2020-15-0201-00608) and performed according to national guidelines. Outbred Rj:SWISS mice (Janvier labs) and timed natural matings were used for all experiments. Noon of the day when the vaginal plugs of mated females were identified was scored as E0.5. E3.25 and E6.25 male and female embryos were collected.

All mouse ESCs used in this study are derivatives of an E14J male line on 129/Ola background. Genetically modified versions of this line are listed in the key resources table. ESCs were maintained in naive conditions on fibronectin-coated (Corning) tissue culture plates in N2B27 media consisting of a 1:1 ratio of DMEM/F12+Glutamax (Gibco) and Neurobasal (Gibco), supplemented with 1xN2 (Gibco), 1xB27 (Gibco), 1mM Glutamax (Gibco) and 100μM b-mercaptoethanol (ThermoFisher Scientific) and 2iLIF: 3μM CT99021 (Axon Medchem), 1μM PD (Axon Medchem) and 10ng/mL LIF (made in house).

### Method details

#### Embryo isolation and culture

E3.25 and E6.25 embryos were collected and morphologically assessed to ensure that only viable samples were collected. Embryos were cultured for five hours at 37°C in 5% CO₂ and 5% O₂ in KSOM medium containing 95 mM NaCl, 2.5 mM KCl, 0.35 mM KH₂PO₄, 0.20 mM MgSO₄, 25 mM NaHCO₃, 1.71 mM CaCl₂, 0.01 mM EDTA, 5.5 mM glucose, 10 mM L-lactate, and 2 mM GlutaMAX. For ^13^C isotope labeling experiments, glucose, lactate or GlutaMAX were replaced with their corresponding [U-^13^C] labeled forms. Following incubation, embryos were washed with PBS supplemented with 0.01% polyvinyl alcohol (PVA), embedded in 10% gelatin, and flash frozen at -80^o^C until cryosectioning.

##### MALDI-MSI Tissue Preparation and Matrix Deposition

Gelatin-embedded embryos were cryosectioned into 10 μm thick sections using a Cryostar NX70 cryostat (Thermo Fisher Scientific, MA, USA) at -20 °C. The sections were thaw-mounted onto indium-tin-oxide (ITO)-coated glass slides (VisionTek Systems Ltd., Chester, UK). Mounted sections were placed in a vacuum freeze-dryer for 15 minutes prior to matrix application. After drying, N-(1-naphthyl) ethylenediamine dihydrochloride (NEDC) (Sigma-Aldrich, UK) MALDI-matrix solution of 7 mg/mL in methanol/acetonitrile/deionized water (70, 25, 5 %v/v/v) was applied using a HTX M3+ Sprayer (HTX Technologies, USA). The setting was as below: temperature, 60 °C; number of passes, 20 layers; flow rate, 80 μL/min; velocity, 2000 mm/min; track spacing, 3 mm; gas flow rate 10 psi, and drying time in between passes, 30 s.

##### MALDI-MSI measurements and data analysis

MALDI-TOF/TOF-MSI was performed using a RapifleX MALDI-TOF/TOF system (Bruker Daltonics GmbH, Bremen, Germany). Negative ion-mode mass spectra were acquired at a pixel size of 5 × 5 μm2 over a mass range from m/z 80-1000. Prior to analysis the instrument was externally calibrated using red phosphorus. Additionally, the laser ablation diameter was confirmed with a single-line laser ablation shot on a matrix-coated tissue section. Spectra were acquired with 30 laser shots per pixel at a laser repetition rate of 10 kHz. Data acquisition was performed using flexControl (Version 4.0, Bruker Daltonics) and visualizations were obtained from flexImaging 5.0 (Bruker Daltonics).

MSI data were exported and processed in SCiLS Lab 2024b pro (SCiLS, Bruker Daltonics) with baseline correction using convolution algorithm. All MALDI-TOF-MSI data were normalized to the Root Mean Square (RMS). Peak picking was performed (signal-to-noise-ratio > 3) on the average spectrum, and matrix peaks were excluded from the m/z feature list. The m/z values from MALDI-TOF were imported into the Human Metabolome Database (https://hmdb.ca/) after re-calibration in mMass and annotated for metabolites with an error < ±20 ppm. The 13C-labeled peaks were selected by comparing the spectrum of control and 13C-labeling experiments and annotated based on the presence of un-labeled metabolites and their theoretical m/z values. Peak intensities of the selected features were exported for all the measured pixels from SCiLS Lab, which were used for the following analysis. Natural isotope abundance correction was performed for metabolites using R package IsoCorrectoR34. Lab, which were used for the following analysis. Natural isotope abundance correction was performed for metabolites using R package IsoCorrectoR34.

For UMAP analysis, the datasets were transformed into a count matrix by multiplying the RMS-normalized intensities by 10 and taking the integer. This count data matrix was normalized and scaled using SCTransform to generate a 2-dimensional UMAP map using Seurat in R (version 4.0). All the datasets were integrated into one dataset after batch correction with rpca method in Seurat. The distribution of the pixels from different clusters on tissues were exported, and cell types were identified based on their morphology. Pixel annotation was consistent across stages and embryos. Clusters specifically associated with extracellular signals were removed from the analysis.

The average values of the exported metabolite in each cell type were calculated. The cell-type specific fraction enrichment of isotopologues was calculated based on the ratio of each 13C-labeled metabolite (isotopologue) to the sum of this metabolite abundance in each cell-type. The calculated fraction enrichment of isotopologues was used to generate pseudo-images together with pixel coordinate information exported from SCiLS Lab.

#### Culture of mouse pluripotent stem cells

For the generation of the E14-DOX-CRISPRi cell line, ESCs were cultured on gelatin-coated tissue culture plates containing Serum/LIF media based on GMEM (Sigma-Aldrich) supplemented with 10% fetal bovine serum (Sigma), 1mM Glutamax (Gibco),1x non-essential AA (Gibco), 1mM sodium pyruvate (Gibco), 100μM b-mercaptoethanol (ThermoFisher Scientific) and 10ng/mL LIF (made in house). EpiLCs and EpiSCs were cultured in FAX conditions on fibronectin-coated tissue culture plates containing N2B27 media supplemented with 20ng/mL Activin A (Qkine), 12ng/mL Fgf2 (Qkine) and 10μM XAV939 (Merck). All cell lines used were maintained under hypoxic conditions (5% O_2,_ 5% CO_2_) at 37°C and were passaged every 2-3 days.

For the ^13^C isotope labeling metabolomic experiments, ESCs were cultured in regular N2B27 supplemented with 2iLIF, and EpiLCs/EpiSCs in N2B27 supplemented with FAX, as described above. Five hours prior cell harvesting, the media were replaced with N2B27 consisted of a 1:1 mixture of DMEM/F12 lacking glucose, glutamine, glutamate and sodium pyruvate (Sartorius/*In vitro*), and Neurobasal lacking glucose and sodium pyruvate. These media were supplemented with either [U-13C]glucose (Merck Sigma-Aldrich), [U-13C] glutamine (Cambridge Isotope Laboratories, Inc.), [1-13C] glutamine (Cambridge Isotope Laboratories, Inc.) or [1-13C] sodium pyruvate (Merck Sigma-Aldrich), together with the corresponding unlabeled compounds and the appropriate signaling factors or inhibitors (2iLIF or FAX).

#### Induction of ESCs to EpiLCs

For metabolomics-related experiments, EpiLCs were generated by washing naive ESCs three times with empty N2B27 and seeded in the appropriate density onto fibronectin-coated plates containing FAX media for 1, 2 or 3 days for the generation of EpiLCs day 1, day 2 or day 3 respectively. For experiments involving the E14-DOX-CRISPRi cell line, cells were first plated in the appropriate density and cultured in 2iLIF in the presence of DMSO or DOX (0.5μg/μl; Merck) for 24 hours. Subsequently, cells were washed three times with empty N2B27 and cultured in FAX conditions for 48 hours with DMSO or DOX. For all the experiments performed, the media was replaced every 24 hours.

#### Differentiation of ESCs towards somatic and extraembryonic lineages

To differentiate ESCs into neuroectoderm and mesoendoderm, we performed monolayer differentiations based on protocols adapted from Mulas et al. (2019)^75^ and Pantier et al. (2021)^45^ (neuroectoderm), and from Mulas et al. (2017)^76^ and Labouesse et al. (2021)^46^ (mesoendoderm). ESCs were washed with PBS, dissociated using Accutase (StemPro) and resuspended in regular N2B27 media consisting of a 1:1 ratio of DMEM/F12+Glutamax (Gibco) and Neurobasal (Gibco), supplemented with 1xN2 (Gibco), 1xB27 (Gibco), 1mM Glutamax (Gibco) and 100μM b-mercaptoethanol (ThermoFisher Scientific) and 2iLIF: 3μM CT99021 (Axon Medchem), 1μM PD (Axon Medchem) and 10ng/mL LIF (made in house). Cells were plated onto laminin-coated plates (10 ug/mL in PBS, Gibco, A29249) and allowed to attach overnight (15 hours). The following day, cells were washed three times with Neuro-N2B27 medium, consisting of a 1:1 mixture of Advanced DMEM/F-12 (Gibco) and Neurobasal (Gibco) supplemented with 1xGlutamax (Gibco), 1xMEM non-essential amino acids (Gibco), 0.5xN2 supplement (Gibco), 0.5xB27 Supplement (Gibco) and 0.1mM 2-mercaptoethanol (Gibco). For neuroectoderm differentiation, cells were continued to be cultures in modified N2B27 for 5, 6, or 7 days, with daily medium changes. For mesoendoderm differentiation, 24 hours after switching to modified N2B27, the media were further supplemented with 20ng/mL Activin A and 3uM CHIR99021, and cells were cultured for an additional 6 days, with daily medium changes. For CRISPRi experiments, doxycycline was added at the time of seeding and replenished daily until cell harvest. All cells were maintained under hypoxic conditions (5% O_2,_ 5% CO_2_) at 37°C.

PrE cells were differentiated from ESCs following Linneberg-Agerholm et al. (2024)^44^ protocol. ESCs (350.000cells) were plated onto 6-well gelatin-coated tissue culture plates for 24 hours in PrE basal medium composed of RPMI 1640 (Gibco), 1xB27 minus insulin (Gibco) and 100μM b-mercaptoethanol (ThermoFisher Scientific). Subsequently, PrE basal media was replaced for RACL medium (PrE basal medium supplemented with 10ng/mL LIF (made in house), 3μM CT99021 (Axon Medchem) and 20ng/mL Activin A (Qkine)) for an additional 6 days. Cells were maintained under hypoxic conditions (5% O_2,_ 5% CO_2_) at 37°C. The media was replenished daily.

#### Intracellular and extracellular metabolite extractions

Prior to cell harvesting, 100 μL of spent growth medium was snap-frozen and stored at −80°C for subsequent extracellular metabolite extraction. Cells were washed three times with PBS, followed by the addition of 1.0 mL ice-cold 90% methanol. For unlabeled metabolomics, methanol was supplemented with isotopically labeled internal standards; for ^13^C isotope tracing, internal standards were omitted. Cells were collected by scraping and stored at −80°C until intracellular metabolite extraction.

For intracellular metabolite extraction, samples were thawed on ice, snap-frozen in liquid nitrogen, thawed again, and vortexed. This freeze–thaw–vortex cycle was repeated three times. Samples were then incubated on ice for 1 hour and centrifuged at 15,000 rpm for 15 minutes at 4°C. For unlabeled metabolomics, 50 μL of the supernatant were transferred to GC vials and dried at room temperature using a nitrogen evaporator set at a flow of 5 L/min. For ^13^C isotope tracing, supernatants were transferred to LC vials and dried at room temperature with a nitrogen evaporator at a flow rate of 10 L/min. Dried extracts were stored at −20°C until further analysis.

For extracellular metabolite extraction, 100 μL of spent growth medium was resuspended in 200 μL of ice-cold 90% methanol containing labeled internal standards. Subsequent steps mirrored those used for intracellular metabolite extraction.

#### Protein extraction and quantification

To normalize unlabeled intracellular and extracellular metabolomics data, total protein content was measured for representative samples. Briefly, cell pellets were obtained by incubating cells with TrypLE for 5 minutes, followed by the addition of growth medium and centrifugation at 1,400 rpm for 5 minutes. The resulting pellets were resuspended in RIPA buffer (50 mM Tris-HCl, pH 7.5; 150 mM NaCl; 1% NP-40; 0.1% SDS; 0.5% sodium deoxycholate; and protease inhibitor cocktail [Sigma-Aldrich, cOmplete]). Cell lysates were incubated on ice for 10 minutes, then sonicated using a Bioruptor Plus (4°C, 8 cycles of 30-second pulses with 30-second intervals). Following sonication, samples were centrifuged at 20,000 × *g* for 10 minutes at 4°C. The supernatant was collected, and protein concentration was quantified using the DC Protein Assay (Bio-Rad, 5000111).

#### Gas chromatography-mass spectrometry (GC-MS) analysis of unlabeled metabolites

GC-MS analysis was performed on a Leco Pegasus BT GC/time-of-flight MS (St. Joseph, MI, United States) coupled to a Gerstel MPS multisampler (Mülheim an der Ruhr, Germany). A two-step derivatization reaction was performed prior to the analysis. In the first step of the reaction- methoximation, 12.5 μL of MOX reagent were added to the dried extracts, and samples were then incubated at 45°C for 1 hour. In the second step of the reaction- trimethylsilylation, methoximated samples were re-incubated with 12.5 μL of BSTFA with 1% TMCS at 45°C for 1 hour. 50 μL of 10 mg/L of 4,4'-dibromooctafluorobiphenyl in hexane were added to the samples to control for precision during injection. A Restek Rxi 5ms column (Bellefonte, PA, United States) with an inlet temperature of 270°C and a helium flow rate of 1.2 mL/min was employed for trimethylsilyl derivatives separation. Temperature gradient started at 40°C for one minute. After, temperature was increased at a rate of 20°C/min until reaching 340°C, where it was maintained for 3 minutes. Mass fragmentation spectra were generated by a 70-eV electron ionization source at an ionization current of 2.0 mA, and it was recorded at 10 Hz in the mass range 50 – 750 m/z. Together with the samples, a series of n-alkanes (C8 – C40) was analysed to calculate retention indexes (RI).

For data processing, raw GC-MS data was converted into centroid mode and exported as netCDF files. Compound identification and peak area extraction was performed with the in-house Swedish Metabolome Centre (SMC; www.swedishmetabolomicscentre.se) GC-MS software and an in-house library based on mass fragmentation spectra and retention indexes of specific compounds. Peak areas were normalized to labeled internal standards.

#### Liquid chromatography-mass spectrometry (LC-MS) analysis of ^13^C labeled metabolites

All LC-MS analyses were performed using a 1290 Infinity II ultra-high-performance liquid chromatography (UHPLC) system (Agilent Technologies, Waldbronn, Germany) coupled to a timsTOF Pro 2 mass spectrometer (Bruker Daltonics, Bremen, Germany).

For central carbon metabolites other than acetyl-CoA, extracted isotope labeled samples were derivatized with 3-nitrophenylhydrazine (3-NPH) according to Hodek et al. (2023),^77^ with modifications. Briefly, 20 μL of 120 mmol L-1 EDC (dissolved in 6% pyridine and 50% methanol) and 20 μL of 200 mmol L-1 3-NPH (dissolved in 50% methanol) were consecutively added to 20 μL of reconstituted samples in 1:1 methanol:water (v/v). The sample was incubated at room temperature (RT) (21°C) for 60 minutes, and afterwards 40 μL of 0.05 mg mL-1 BHT (dissolved in 30% pure methanol) were added to the samples and vortexed. 2 μL of each sample were analyzed on an Acquity UPLC HSS-T3 column (100 × 2.1 mm, 1.8 μm, Waters, MA, USA) by using gradient elution of 0.1% formic acid (v/v) in water as mobile phase A and 0.1% formic acid in acetonitrile as mobile phase B. The flow rate was set at 0.35 mL min-1 with the following gradient: 0 min (5% B), 12 min (100% B), 13 min (100% B), 14 min (5% B), 17 min (5% B). Column and autosampler were kept at 30 °C and 4 °C, respectively.

For acetyl-CoA analysis, 100 μL of a sample extract was evaporated and redissolved in 20 μL of 50% MeOH, and thereafter 2 μL was injected onto an Acquity Premier BEH-Amide VanGuard 50 × 2.1 mm, 1.7 μm column (Waters, MA, USA). The mobile phases were: (A) 10 mM ammonium acetate in water with 5 μM medronic acid, and (B) 10 mM ammonium acetate in 90% aqueous acetonitrile with 5 μM medronic acid. The column was operated at 0.35 mL/min using the following gradient: 0 min (95% B), 5min (60% B), 7 min (30% B), 3 min (30% B), 8 min (30% B), and 8.5 min (95% B). Column and autosampler temperatures were maintained at 40 °C and 4 °C, respectively.

For the MS analysis of all metabolites, the mass spectrometer was operated in negative ionization mode. Ions were generated with a vacuum insulated probe heated-electrospray ionisation (VIPHESI) source. Detection of the mass/charge ratio (m/z) of ions was set from 50 to 1000, and acquisition rates were set to 2 Hz, and the resolution was approximately 60,000. For mass calibration 50 μL internal calibrant of 10 mM Na-formate was injected at the beginning of each analysis. Data acquisition was performed with Control version 6.0 and Bruker Compass HyStar version 5.0 (Bruker Daltonics, Bremen, Germany). Data processing and calculation of isotopologue enrichment was performed with Bruker TASQ 2023b.

Total carbon contribution was calculated using the following equation^78^:

$$\frac{\sum_{i=0}^{n}i\times mi}{\left(n\times \sum_{i=0}^{n}mi\right)}\text{,}$$

where *n* denotes the total number of carbons in the metabolite, *i* represents the different mass isotopologues and *m* corresponds to the abundance of a certain mass.

#### Generation of E14-DOX-CRISPRi cell line

E14 ESCs grown in serum/Lif were nucleofected using the P3 Primary Cell 4D-Nucleofector X (Lonza) with the following 3 plasmids: px459 plasmid containing Cas9 and a sgRNA targeting the *Tigre* locus, the *pCAG-TetON3G* plasmid and *pTetO-dCas9-KRAB-MeCP2-HaloTag* plasmid. Cells resulting from this transfection were subjected to blasticidin (10μg/mL; Merck) and puromycin (1μg/mL; Merck) antibiotic selection for 5 days. Subsequently, colonies were picked, grown and treated with DOX (0.5μg/μl; Merck) for 24 hours. Next, cells were incubated with the HaloTag ligand Janelia Fluor®646 (Promega) and analyzed by flow cytometry to determine expression of dCas9-KRAB-MeCP2. The chosen clone was further validated by immunostaining and immunoblotting and retained a normal karyotype.

#### Cloning of sgRNAs

The individual sgRNAs used for this study were cloned into a PiggyBac vector following the original protocol.^79^^,^^80^ Muti-sgRNAs used for the generation of the Neg, 3xKD, 4xQKD and 4xTKD cell lines were cloned into a modified version of the original piggyBac vector following a published protocol.^81^ The sgRNAs used for this study target the gene’s transcriptional start site and are listed in Table S4.^82^

#### Transfection of sgRNAs into E14-DOX-CRISPRi cells

ESCs were seeded onto 12-well tissue culture plates. The next day, mix A (75μl OptiMem (ThermoFisher Scientific) + 5μl lipofectamine 2000 (ThermoFisher Scientific)) and mix B (75μl OptiMem + 1μg of PiggyBac vector harboring the sgRNA(s) of interest +0.5μg of pBASO) were prepared and incubated separately for 5min at RT. Thereafter, mix A and B were combined and incubated for 15min at RT. The transfection mix was added onto the cells directly drop-by-drop. After 8 hours the media was refreshed and selection with hygromycin (160μg/mL; ThermoFisher Scientific) was started.

#### Generation of IDH-AID-GFP degron cell line

E14 ESCs grown in serum/Lif were nucleofected using the P3 Primary Cell 4D-Nucleofector X (Lonza) with the following 2 plasmids: the idh1-aid-gfp targeting plasmid and the px459 plasmid containing a Cas9 and a sgRNA targeting the 3’ end of the *idh1* gene. Cells resulting from this transfection were subjected to blasticidin (10μg/mL; Merck) antibiotic selection for 5 days. Subsequently, colonies were picked, grown and PCR genotyped for the correct insertion of the two homology arms (HA) and the absence of a WT band. Additionally, the DNA of selected clones was sequenced at the 5’ and 3’ of the insertion to ensure the absence of mutations. The selected clone was validated and karyotyped, and thereafter nucleofected following the same procedure with two additional plasmids: the pCAG-Tir1-Arf16 plasmid and the px459 plasmid containing a Cas9 and a sgRNA targeting the *Tigre* locus. Cells were subjected to puromycin (1μg/mL; Merck) antibiotic selection for 5 days and colonies were picked, grown and PCR genotyped. Functionality of TIR1 was directly assessed on clones B5 and F2 after karyotyping by assessing GFP signal loss upon IAA treatment (250μM; Merck).

#### RNA extraction and RT-qPCR

Total RNA was extracted using Qiagen RNeasy Mini Kit (Qiagen) with a 15min treatment of RNAse-free DNAse (Qiagen) at RT. cDNA was synthesized using Superscript III Reverse Transcriptase (ThermoFisher Scientific) following the manufacturers protocol. Subsequently, RT-qPCR was performed using PowerUp™ SYBR™ Green Master Mix (Thermo Fisher Scientific) in a LightCycler480 (Roche). Primers used for the analysis are listed in Table S4.

#### Immunoblotting

For the obtention of whole cell lysates, cells were harvested and lysed in RIPA buffer (10mM Tris pH7.4, 100mM NaCl, 1mM EDTA, 1mM EGTA, 1mM NaF, 20mM Na4P2O7, 2mM Na3VO4, 1% Triton X-100, 10% glycerol, 0.1%SDS and 0.5% deoxycholate) supplemented with proteinase inhibitor cocktail tablet (Roche) and phosphatase inhibitors (Roche). Lysates were sonicated for 8 cycles (30s ON, 30s OFF). Protein quantification was performed with the Pierce^TM^ BCA protein assay kit (ThermoFisher Scientific). Protein samples were boiled in NuPAGE™ LDS Sample Buffer 4x (ThermoFisher Scientific) with 10mM dTT (ThermoFisher Scientific) at 70°C and separated in a 4-12% NuPage Bis-Tris gel (ThermoFisher Scientific). Proteins were transferred into a nitrocellulose membrane (Merck), which was blocked with 5% skimmed milk in PBST (PBS with 0.1% Tween-20) for at least 1h at RT. Subsequently, the membranes were incubated with the primary antibody overnight at 4°C. On the following day, membranes were washed three times with PBST for 10min and incubated with the corresponding HRP-conjugated secondary antibodies for 1h at RT. Thereafter, membranes were washed and developed using ECL^TM^Select western blotting detection reagent (Merck) using ChemiDoc MP Imaging System (BioRad). The intensity of the bands was quantified by ImageJ 1.54f.

#### Immunostaining

Cells were cultured in 96-well imaging plates (PerkinElmer), washed with PBS and fixed with 4%formaldehyde (ThermoFisher Scientific) for 15min at RT. Subsequently, cells were washed twice with PBS, permeabilized with permeabilization buffer (0.5% Triton X-100 in PBS) for 15min at RT and blocked with blocking buffer (10% goat serum, 1% bovine serum albumin, 0.1%Tween in PBS) overnight at 4°C. Primary antibodies were diluted in blocking buffer and incubated overnight at 4°C. Next day, cells were washed three times with PBST (0.1% Tween20 in PBS) for 10min and incubated with secondary antibodies diluted in blocking buffer overnight at 4°C. Thereafter, cells were washed once with PBST and stained with DAPI (0.5μg/mL, Sigma-Aldrich) for 15min at RT. Finally, cells were washed three times with PBS before proceeding to microscope imaging and downstream analysis.

Embryos were isolated at E3.5 and E6.5 and the Reichert’s membrane and visceral endoderm was removed as described previously.^83^ The embryos were subsequently fixed in 4% PFA with 0.001% PVA for 20 min at 37 °C, then washed 3 times in PBS-T (0.1% Tween-20, 0.001% PVA) for 10 min at room temperature (RT). Permeabilization was performed for 20 min in 0.1 M glycine, 0.5% Triton X-100, and 0.001% PVA, followed by three additional PBS-T washes. Embryos were blocked overnight at 4 °C in blocking buffer (1% BSA, 0.1% Tween-20, 10% normal goat serum, 0.001% PVA in PBS), then incubated with primary antibodies (in blocking buffer) for 1–2 days at 4 °C, rocking. After washing (3 times, 10 min each, PBS-T - 0.001% PVA), embryos were incubated with secondary antibodies overnight at 4 °C in the dark. Embryos were washed (3 times, 30 min each, PBS-T - 0.001% PVA - DAPI was added during the second wash step at a final concentration of 1μg/ml) and mounted on glass-bottom plates in SlowFade™ Diamond Antifade Mountant (Invitrogen).

#### Janelia Fluor staining and Flow cytometry

The HaloTag ligand Janelia fluor® 646 (200nM, Promega) was added to the cell culture media and cells were incubated for 1h under regular cell culture conditions. Thereafter, cells were washed two times with PBS, incubated in clean media for 15 min and harvested for flow cytometry analysis. Cytek® Aurora Spectral Flow Cytometer was used for flow cytometry and the data was processed and analyzed using the FCS Express 7 software (De Novo Software).

#### Karyotyping

Cells were plated onto gelatin-coated 10cm dishes and treated with colcemid (0.1μg/mL, ThermoFisher Scientific) for 3 hours. Subsequently, cells were gently harvested and pelleted at 300g for 3 min and resuspended in 100μl of PBS. Next, 10mL of pre-warmed 75mM KCl was added drop by drop by pipetting to the wall of the tube and incubated for 15 min at 37°C. Thereafter, cells were spun down for 5 min at 300g using a faced angle rotor. The buffer was removed and freshly prepared fixation buffer (3:1 absolute methanol to glacial acetic acid) was added using a glass Pasteur pipette drop by drop while gently vortexing the tube. Cells were incubated at 4°C overnight. The next day, the fixation buffer was removed, leaving behind around 500mL that was used to resuspend the fixed cells. To prepare the metaphase spreads, 100μl was dropped onto a washed slide from around 20cm by tapping the pipette with force. Slides were dried for at least 30min and subsequently stained with 5% Giemsa solution (ThermoFisher Scientific) for 30 min at RT. Finally, the slides were rinsed twice with deionized water and left to dry for at least 30 min at RT. Samples were mount with 100μl of DPX mounting media (Merck).

#### Bulk RNA-seq

E14-DOX-CRISPRi (Neg and 3xKD) cells were seeded in the appropriate density onto 6-well tissue culture fibronectin-coated plates in N2B27+2iLif with DMSO or DOX for 24 hours. ESCs were maintained for 48 hours more in the same conditions. For the generation of EpiLCs day 2, cells were washed three times with empty N2B27 and subsequently were cultured in FAX conditions with DMSO or DOX for 48 hours. The media was exchanged every 24 hours for all the cell lines and conditions. Puromycin (1μg/mL; Merck), blasticidin (10μg/mL; Merck) and hygromycin (160μg/mL; ThermoFisher Scientific) selection was kept throughout the length of the experiment. All cells were harvested in parallel for downstream processing. RNA was purified using the RNeasy Mini Kit (Qiagen) with a 15min treatment of RNase-Free DNase (Qiagen) at RT. 500 ng of RNA per sample was used for library preparation with the NEBNext rRNA Depletion Kit v2 (New England Biolabs), NEBNext Ultra II Directional RNA Library Prep Kit for Illumina (New England Biolabs) and NEBNext Multiplex Oligos for Illumina Dual Index Primers Set 2 (New England Biolabs). The quality of the RNA and cDNA was determined using a TapeStation (Agilent). Barcoded libraries were pooled in equimolar amounts and subjected to single-end sequencing on the Illumina NextSeq 2000 sequencer. Raw sequencing data were demultiplexed and converted into FASTQ files using Illumina bcl2fastq Conversion Software (v2.20.0.422). Resulting FASTQ files were processed using nf-core/rnaseq v3.14.0 of the nf-core collection of workflows.^84^ In brief, adapters and low-quality reads were removed using Trim Galore (v0.6.7), trimmed reads were mapped to the mouse reference genome (GRCm39, Ensembl release 111) using STAR (v2.7.9a) and transcript expression was quantified with Salmon (v1.10.1).^85^^,^^86^ Transcript-level abundance estimates were imported into R (v4.3.2) and summarized to gene level using tximport package (v1.30.0).^87^ A minimal pre-filtering step was applied to retain genes with at least 10 total reads.

Differential gene expression analysis was conducted using DESeq2 (v1.28.0).^88^ Genes were considered significantly differentially expressed if they met the following criteria: an adjusted *p*-value < 0.01 and an (absolute(log_2_(fold change)) > 0.58 across the specified comparisons. Gene set enrichment analysis for the significantly upregulated and downregulated genes in the 3xKD versus Neg in EpiLCs was performed using the clusterProfiler (v4.10.1) package and visualized with the GseaVis (v0.0.5) package.^89^^,^^90^

For *K*-means clustering, the likelihood ratio test (LRT) from the DESeq2 package was used to identify differentially expressed genes, with significance defined as an adjusted *p*-value < 0.01. *K*-means clustering was performed on the resulting matrix of rlog transformed counts using the pheatmap package (v1.0.12). The same package was utilized to visualize the results as heatmap. Gene Ontology (GO) and KEGG pathway enrichment analyses were performed for each gene cluster using the clusterProfiler package and/or Cytoscape.^91^ The analysis focused on the Biological Process ontology and KEGG pathways, with significance defined as an adjusted *p*-value < 0.01.

#### Colony formation assay and alkaline phosphatase staining

EpiLCs day 2 were generated as described above. Cells were washed thoroughly with N2B27 three times and seeded at low density in naive culture conditions. Five days later, colonies were fixed and stained with leukocyte alkaline phosphatase kit (Merck) following the manufacturer’s protocol. AP+ colonies were imaged using GelDoc XR^+^(BioRad), identified and quantified by ImageJ 1.54f.

#### Histone enrichment and quantification of histone PTMs by MS

ESCs, EpiLCs day 2 and EpiSCs were cultured for 48 hours under the corresponding media conditions and as described above. 5 hours prior harvesting, the media was replaced with fresh regular media (for the unlabeled samples) or with N2B27 depleted of glucose or glutamine and supplemented with [U-13C]glucose (Merck Sigma-Aldrich) or [U-13C]glutamine (Cambridge Isotope Laboratories, Inc.) respectively. Thereafter, cells were resuspended vigorously in 1ml of nuclei isolation buffer (0.5mM PMSF, 5μg/ml Leupeptin (Merck Sigma-Aldrich), 5μg/ml Aprotinin (Merck Sigma-Aldrich), 5mM Na-butyrate, 0.1% Triton) to facilitate rupture of the membranes. The nuclei were isolated by centrifugation for 15 min at 2300g at 4°C. Next, the nuclear pellet was resuspended with 100μl of nuclei isolation buffer supplemented with 0.1% SDS and 250 U of benzonase (Merck Sigma-Aldrich) and incubated for 5 min at 37°C to digest nuclear extracts. Protein concentration was measured using DC Protein Assay (Bio-Rad, 5000111).

Approximately 4 mg of histone octamer were mixed with an approximately equal amount of a heavy-isotope labeled histone standard and were separated on a 17% SDS-PAGE gel.^92^ Histone bands were excised, chemically acylated with propionic anhydride and in-gel digested with trypsin, followed by peptide N-terminal derivatization with phenyl isocyanate (PIC)^93^ Peptide mixtures were separated by reversed-phase chromatography on an EASY-Spray column (Thermo Fisher Scientic), 25-cm long (inner diameter 75 μm, PepMap C18, 2 μm particles), which was connected online to a Q Exactive Plus instrument (Thermo Fisher Scientific) through an EASY-Spray™ Ion Source (Thermo Fisher Scientific), as described.^93^ The acquired RAW data were analyzed using Epiprofile 2.0,^94^ selecting the “histone_C13” and “SILAC” options, followed by manual validation.^93^ For each acetylated peptide, a % 13C relative abundance (%RA) was estimated by dividing the area under the curve (AUC) of 13C-labeled peptides for the sum of the areas corresponding to all the observed labeled/unlabeled forms of that peptide and multiplying by 100. For the experiment shown in Figure S4G, the levels of common histone acetylations and methylations were quantified as described.^93^ The mass spectrometry data have been deposited to the ProteomeXchange Consortium^95^via the PRIDE partner repository with the dataset identifier PXD062776.

#### Image analysis and quantification

Images of the immunostainings (Figures 6B, 6C, 6H and S6J) were acquired using an Olympus IXplore Spinning Disk Confocal microscope using an Olympus UPLXAPO 20x0.80– WD 0.60mm objective. For each well in a 96-well plate, 12 images composed of 30 Z-fields separated by 1μm each were taken. The ScanR analysis software (version 3.2.0, r4066 x64, Olympus) was used for image segmentation and quantification of the following parameters: X, Y, area, well, circularity, median, average and total fluorescent intensities of the corresponding antibodies. Images of the immunostainings (Figures 6D, 6F, 6G, 6J, S6E, and S6I) were acquired using the Opera Phenix high content spinning disk confocal microscope using a 20xWater NA1.0 W Plan Apochromat/ WD 1.7mm objective. For each well in a 96-well plate, 9 images composed of 35 Z-fields separated by 1μm each were taken. The Harmony high content imaging and analysis software was used for image segmentation and quantification of the following parameters: X, Y, well, object number, mean, median, average and total fluorescent intensities of the corresponding antibodies. Subsequently, the data was imported to the program TIBCO® Spotfire for further analysis. The imaging of mouse embryos was performed using a Leica SP8 Confocal microscope.

### Quantification and statistical analysis

All the experiments were performed at least in biological triplicates (*n* = 3), unless otherwise stated in the legend. The number of biological and technical replicates and the number of embryos are indicated in figure legends. One embryo is considered a biological replicate; all embryo experiments originate from at least two litters. Sample size was not predetermined. For all experiments with error bars, standard error of the mean (SEM) was calculated to indicate the variation within each experiment or sample. The statistical tests were performed using R and GraphPad Prism 10. The statistical details for each experiment can be found in the figure legends. In Figures 2–6, Levene's test was used to evaluate homogeneity of variances, and Shapiro-Wilk test was applied to assess the normality of residuals for each metabolite.

### Additional resources

There are no additional resources linked to this publication.

## Supplemental information

Document S1. Figures S1–S6

Table S1. Metabolomic datasets, related to Figures 1, 2, 3, 4, 5, and 6 ^13^C metabolite labeling and metabolite abundances in embryos, stem cells and following the repression of the genes *Pcx*, *Me1*, *Me2*, and all three (3×KD) in ESCs and EpiLCs.

Table S2. Gene ontology analysis, related to Figure 3 *K*-means clusters with Gene Ontology (GO) analysis for the transcriptional changes in 3×KD versus Neg in ESCs and EpiLCs day 2. Only the top GO terms are shown.

Table S3. Differential gene expression analysis, related to Figure 3 DSeq2 analysis for the transcriptional changes in 3×KD versus Neg in ESCs and EpiLCs day 2. Only the statistically significant results (adjusted *p* value < 0.01 and a log_2_ transformed fold change > 0.58 or < −0.58) for the different comparisons are shown.

Table S4. Oligonucleotides and sgRNAs used for this study, related to Figures 3 and 6

Document S2. Article plus supplemental information
