# Supplementary material for: TCA cycle rewiring underpins histone acetylation sourcing and cell-fate transitions during exit from naive pluripotency
Source: Cell Stem Cell. 2026 May 7;33(5):820–836.e9. doi: 10.1016/j.stem.2026.04.004 (PMC13174499; doi:10.1016/j.stem.2026.04.004)
Supplement: Document S1. Figures S1–S6 [file mmc1.pdf]

Cell Stem Cell, Volume *33*

Supplemental Information

TCA cycle rewiring underpins histone acetylation sourcing and cell-fate transitions during exit from naive pluripotency

Eleni Kafkia, David Pladevall-Morera, Lidia Argemi-Muntadas, Gangqi Wang, Roberta Noberini, Arnau Casòliba-Melich, Sandra Bagés-Arnal, Matthias Anagho-Mattanovich, Rita Silvério-Alves, Johanna Gassler, Tiziana Bonaldi, Ton J. Rabelink, Thomas Moritz, and Jan Jakub Żylicz

Supplementary figures

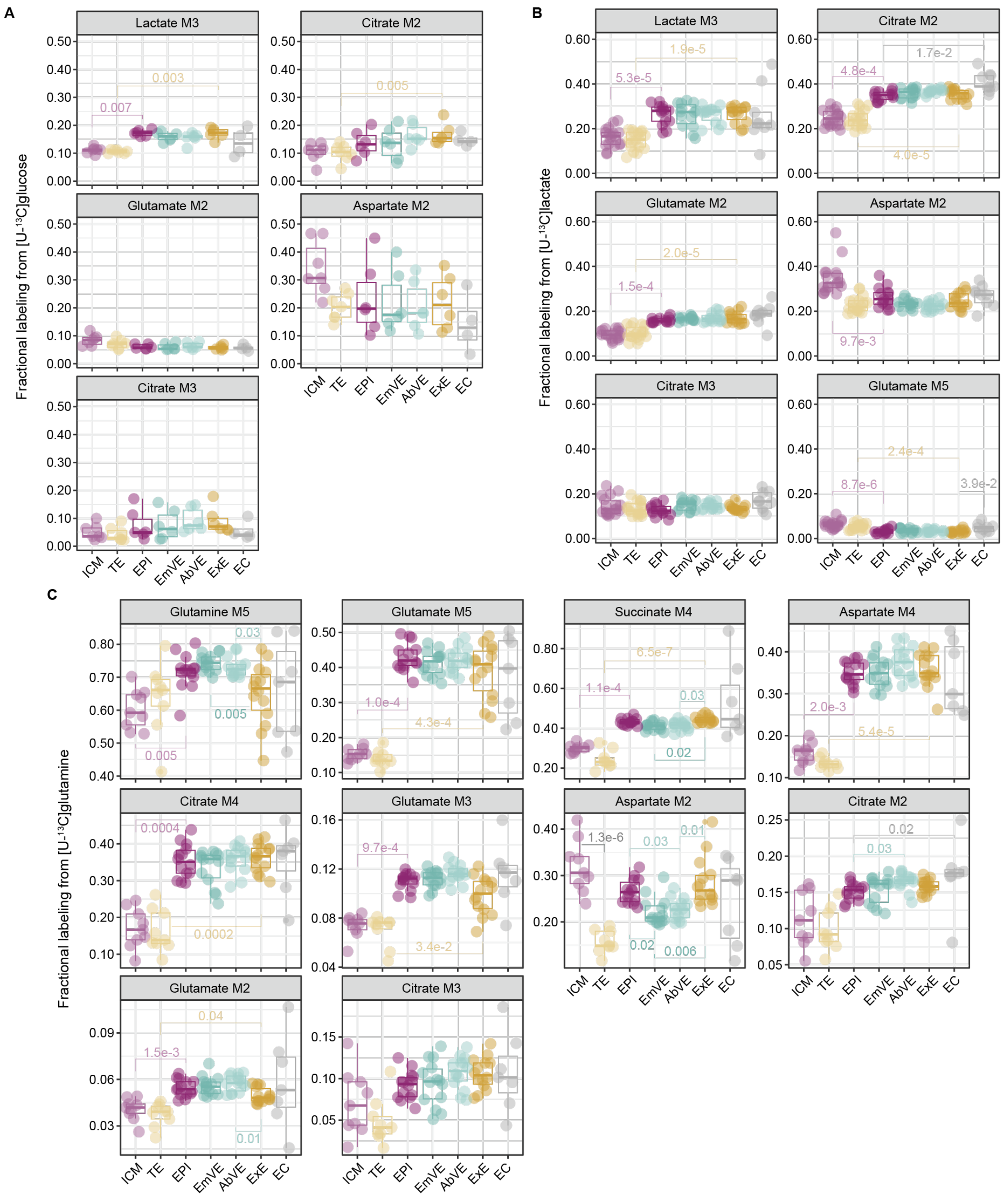

Supplementary Figure 1. Fractional labeling of metabolites in E3.5 and E6.5 embryos - Related to Figure 1

(**A-C**) Fractional labeling of metabolites derived from [U-^{13}C]glucose (A), [U-^{13}C]lactate (B) or [U-^{13}C]glutamine (C) in spatially resolved lineages in E3.5 and E6.5 embryos. (A) ICM, n = 7; TE, n = 7; EPI, n = 6; EmVE, n = 6; AbVE, n = 6; ExE, n = 6; EC, n = 4. (B) ICM, n = 18; TE, n = 18; EPI, n = 14; EmVE, n = 14; AbVE, n = 14; ExE, n = 14; EC, n = 9. (C) ICM, n = 9; TE, n = 9; EPI, n = 14; EmVE, n = 14; AbVE, n = 14; ExE, n = 14; EC, n = 7. ICM, inner cell mass; TE, trophectoderm; EPI, epiblast; EmVE, embryonic visceral endoderm; AbVE, abembryonic visceral endoderm; ExE, extraembryonic ectoderm; EC, ectoplacental cone. Data points correspond to individual embryos. Statistical significance was assessed using the Kruskal-Wallis test followed by Dunn's post-hoc test, with p-values adjusted with the Benjamini-Hochberg (BH) correction method.

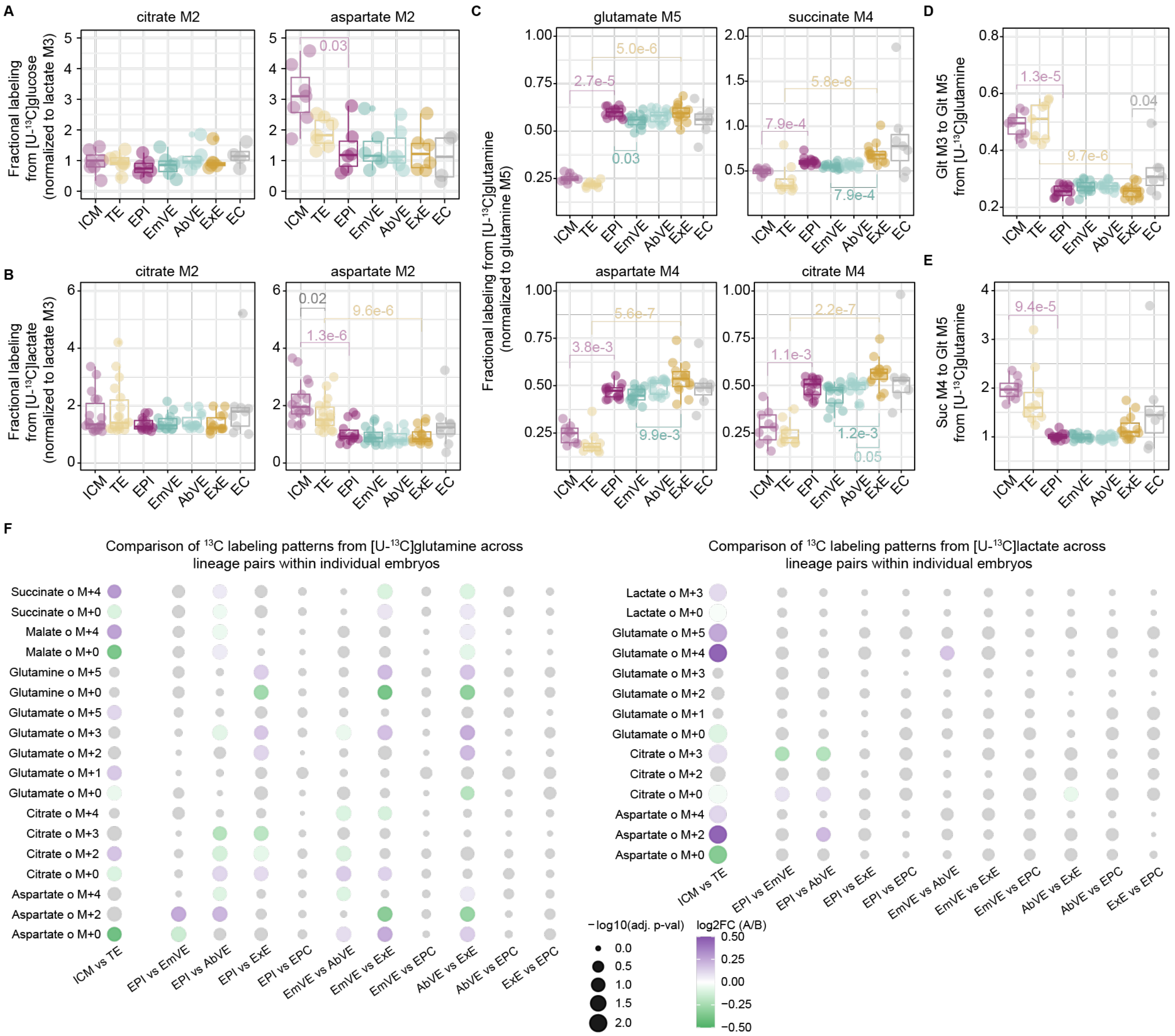

Supplementary Figure 2 – Relative fractional labeling of metabolites in E3.5 and E6.5 embryos - Related to Figure 1

(**A-C**) Fractional labeling of metabolites derived from [U-^{13}C]glucose (A) [U-^{13}C]lactate (B) and [U-^{13}C]glutamine (C) in spatially resolved lineages in E3.5 and E6.5 embryos. The values are normalized to M3 lactate (A,B) and M5 glutamine (C). (A) ICM, n = 7; TE, n = 7; EPI, n = 6; EmVE, n = 6; AbVE, n = 6; ExE, n = 6; EC, n = 4. (B) ICM, n = 18; TE, n = 18; EPI, n = 14; EmVE, n = 14; AbVE, n = 14; ExE, n = 14; EC, n = 9. (C) ICM, n = 9; TE, n = 9; EPI, n = 14; EmVE, n = 14; AbVE, n = 14; ExE, n = 14; EC, n = 7. (D) Fractional labeling M3 glutamate normalized to M5 glutamate from [U-^{13}C]glutamine in spatially resolved lineages in E3.5 and E6.5 embryos. ICM, n = 9; TE, n = 9; EPI, n = 14; EmVE, n = 14; AbVE, n = 14; ExE, n = 14; EC, n = 7. (E) Fractional labeling of M4 succinate normalized to M5 glutamate from [U-^{13}C]glutamine in spatially resolved lineages in E3.5 and E6.5 embryos. ICM, n = 9; TE, n = 9; EPI, n = 14; EmVE, n = 14; AbVE, n = 14; ExE, n = 14; EC, n = 7. (F) Comparison of ^{13}C labelling patterns from [U-^{13}C]glutamine (left) and [U-^{13}C]lactate (right) across lineage pairs within individual embryos. Dot size reflects the –log10 of the adjusted p-value for per-embryo fractional labeling of metabolites across all lineage pair comparisons. Color indicates the average log_2 fold change in fractional labeling of metabolites across all lineage pair comparisons. Only dots with an adjusted p-value<0.05 are colored. ICM, inner cell mass; TE,

trophectoderm; EP**I**, epiblast; EmVE, embryonic visceral endoderm; AbVE, abembryonic visceral endoderm; ExE, extraembryonic ectoderm; EC, ectoplacental cone. (A-E) Data points correspond to individual embryos. Statistical significance was assessed using the Kruskal-Wallis test followed by Dunn's post-hoc test, with p-values adjusted with the Benjamini-Hochberg (BH) correction method. (F) Statistical significance was assessed using Paired Wilcoxon signed-rank test with BH correction where fractional labeling values in individual lineages were paired within each embryo analyzed. (**A-E**) ICM, inner cell mass; TE, trophectoderm; EP**I**, epiblast; EmVE, embryonic visceral endoderm; AbVE, abembryonic visceral endoderm; ExE, extraembryonic ectoderm; EC, ectoplacental cone. (A-E) Data points correspond to individual embryos. Statistical significance was assessed using the Kruskal-Wallis test followed by Dunn's post-hoc test, with p-values adjusted with the Benjamini-Hochberg (BH) correction method.

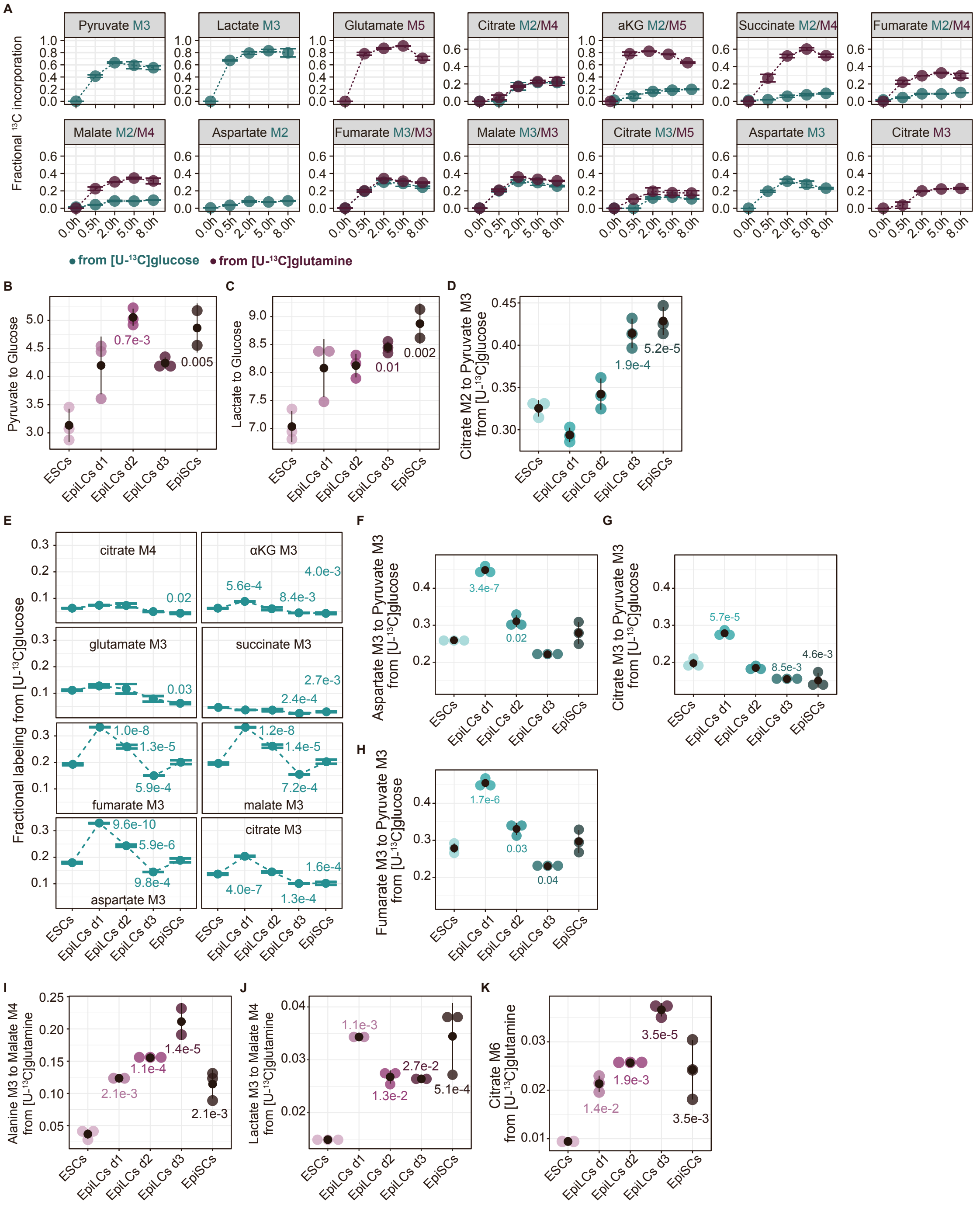

Supplementary Figure 3. Fractional labelling of TCA cycle intermediates in pluripotent stem cells - Related to Figure 2

(**A**) Fractional labelling of TCA cycle intermediates in EpiSCs following supplementation with [U-^{13}C]glucose (green) or [U-^{13}C]glutamine (purple) for the indicated time points. Specific isotopologues are displayed. (**B-C**) Ratio of pyruvate (**B**) and lactate (**C**) to glucose based on intracellular metabolite abundances throughout pluripotency progression (n = 3). (**D**) Relative glucose entry to the TCA cycle through the activity of pyruvate dehydrogenase throughout pluripotency progression, represented by the ratio of M2 citrate to M3 pyruvate

from [U-^{13}C]glucose (n = 3). (**E**) Fractional labelling of TCA cycle intermediates from [U-^{13}C]glucose throughout pluripotency progression, focusing on isotopologues derived from the second turn of the cycle (n = 3). (**F-H**) Relative pyruvate carboxylase activity throughout pluripotency progression, represented by the fractional labeling of M3 isotopologues of aspartate (F), citrate (G) and fumarate (H) normalized to M3 pyruvate from [U-^{13}C]glucose (n = 3). (**I-J**) Relative malic enzyme activity throughout pluripotency, represented by the fractional labeling of M3 isotopologues of alanine (I) and lactate (J) normalized to M4 malate from [U-^{13}C]glutamine (n = 3). (**K**) Fractional labeling of M6 citrate from [U-^{13}C]glutamine throughout pluripotency (n = 3). (A) Data represent the mean of biological replicates ± standard error of the mean (SEM) per condition. (B-K) Data represent the mean of three biological replicates ± standard deviation (SD) (B-D,F-K) or ± standard error of the mean (SEM) (E) per condition, with each colored point indicating an individual biological replicate. Statistical significance was assessed using one-way ANOVA followed by Tukey's HSD post-hoc test. Levene's test was used to evaluate homogeneity of variances, and Shapiro-Wilk test was applied to assess the normality of residuals for each metabolite.

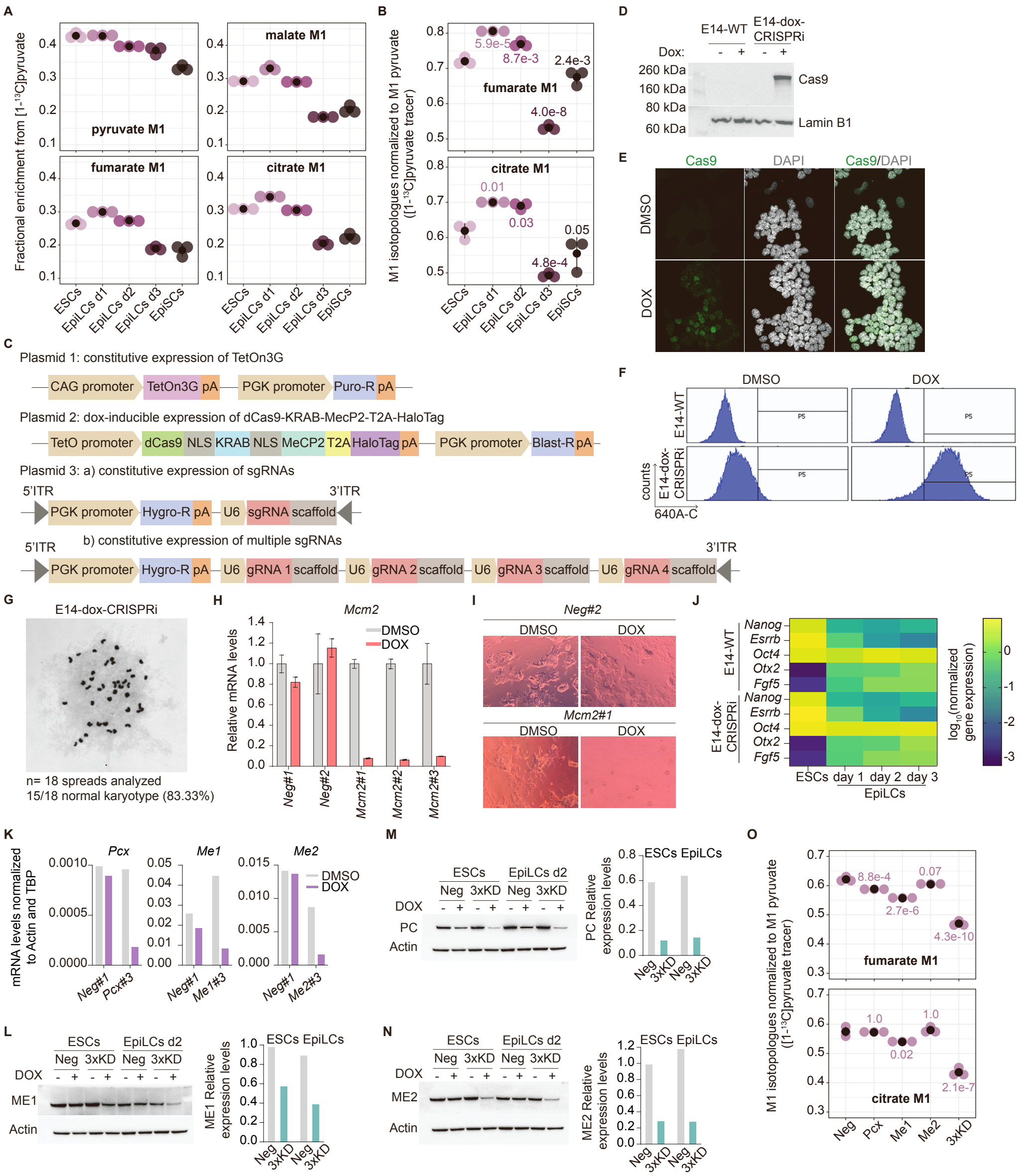

Supplementary Figure 4 – Functional assessment of metabolic enzymes using CRISPRi – Related to Figure 2 and 3

(**A**) Fractional labelling of TCA cycle intermediates from [1-^{13}C]pyruvate throughout pluripotency progression (n = 3). (**B**) Relative pyruvate carboxylase activity throughout pluripotency progression, represented by the fractional labeling of M1 isotopologues of fumarate (upper panel) and citrate (lower panel) normalized to M1 pyruvate from [1-^{13}C]pyruvate (n = 3). (**C**) Plasmids employed for the generation of the E14-DOX-CRISPRi cell line. Plasmid 1: Integrated into one *Tigre* allele for constitutive expression of the TetOn3G DOXycycline(DOX)-binding transactivator protein under the control of the CAG promoter. This plasmid also includes a puromycin resistance cassette driven by the PGK promoter. Plasmid 2: Integrated into the other *Tigre* allele. Upon DOX treatment, TetOn3G binds to the TetO promoter, inducing transcription of the dCas9-KRAB-MeCP2-T2A-HaloTag. The T2A self-cleaving peptide facilitates the separation of dCas9-KRAB-MeCP2 and HaloTag proteins after translation. Additionally, this plasmid contains a

blasticidin resistance cassette driven by the PGK promoter. Plasmid 3: Represents the piggyBAC plasmids, with plasmid 3a encoding a single sgRNA, and plasmid 3b accommodating up to four sgRNAs. Constitutive expression of sgRNA(s) guides the dCas9-KRAB-MeCP2 complex to the transcriptional start site of the target genes. Plasmid 3 also harbors a hygromycin resistance cassette under the PGK promoter. (**D**) Immunoblot analysis of whole-cell lysates from E14-WT and E14-DOX-CRISPRi ESCs following 24-hour treatment with either DMSO or DOX. Samples were probed with Cas9 and Lamin B1 antibodies. (**E**) Immunofluorescence analysis of E14-DOX-CRISPRi ESCs after 24 hours of treatment with either DMSO or DOX. Cells were stained with a Cas9 antibody (green). (**F**) Flow cytometry histogram analysis of Janelia Fluor 646 (HaloTag ligand) in E14-WT and E14-DOX-CRISPRi ESCs after 24 hours of treatment with DMSO or DOX. (**G**) Bright field image of a chromosome spread used for karyotyping the E14-DOX-CRISPRi cell line. Analysis of 18 metaphase spreads revealed that 83.33% exhibited a normal karyotype of 40 chromosomes. (**H**) RT-qPCR analysis of *Mcm2* expression in the indicated cell lines following 72 hours of DMSO (gray) or DOX (red) treatment. mRNA levels were normalized to the expression of the housekeeping genes *actin* and *tbp*. (**I**) Representative bright-field images of E14-DOX-CRISPRi cells transfected with either *Neg#2* or *Mcm2#1* sgRNAs following 5 days of DMSO or DOX treatment. (**J**) Heatmap displaying log_{10}-normalized gene expression of key pluripotency genes in E14-WT and E14-DOX-CRISPRi ESCs and EpiLCs (day 1 to day 3). (**K**) RT-qPCR analysis of the indicated genes in the specified cell lines following 72 hours of DMSO (gray) or DOX (purple) treatment. mRNA levels were normalized to the expression of housekeeping genes *actin* and *tbp*. (**L-N**) Immunoblot analysis of whole-cell lysates from E14-DOX-CRISPRi (Neg and 3xKD) ESCs and EpiLCs (day 2) following 72 hours of DMSO or DOX treatment. Blots were probed with antibodies for PC, ME1, ME2 and actin. The intensity of the bands was measured using ImageJ 1.54f. The levels of PC, ME1 and ME2 were normalized to the respective actin loading control. Bar plots show the relative expression levels of DOX-treated samples compared to their respective DMSO counterparts. (**O**) Relative pyruvate carboxylase activity in EpiLCs day 1 in scramble control (Neg) cells and following the repression of *Pcx*, *Me1*, *Me2*, and all three genes (3xKD). Activity is represented by the fractional labeling of M1 isotopologues of fumarate (upper panel) and citrate (lower panel) normalized to M1 pyruvate from [1-^{13}C]pyruvate (n = 3). (A, B, O) Data represent the mean of biological replicates ± standard error of the mean (SEM) per condition, with each colored point indicating an individual biological replicate. (B, O) Statistical significance was assessed using one-way ANOVA followed by Tukey's HSD post-hoc test. Levene's test was used to evaluate homogeneity of variances, and Shapiro-Wilk test was applied to assess the normality of residuals for each metabolite.

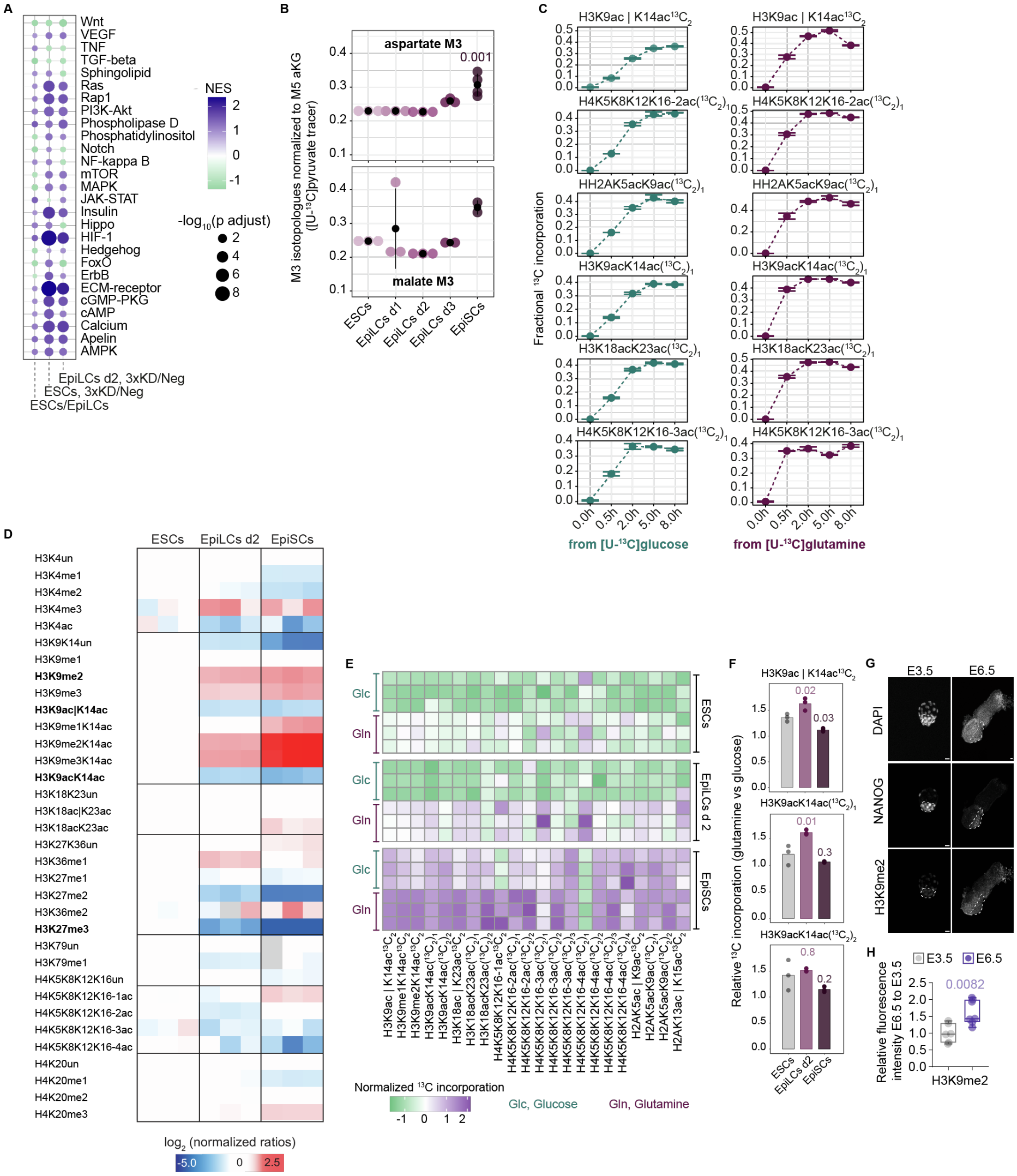

Supplementary Figure 5. Chromatin changes at the exit from naive pluripotency – Related to Figure 3, 4 and 5

(A) Gene set enrichment analysis of significantly differentially expressed genes, focusing on signaling pathways from the KEGG database. Comparisons include Neg ESCs versus Neg EpiLCs (column 1), 3xKD versus Neg in ESCs (column 2), and 3xKD versus Neg in EpiLCs day 2 (column 3). The normalized enrichment score (NES) is represented by a color gradient, while the -$\log_{10}$transformed adjusted *p* values are indicated by circle size. (**B**) Fractional labeling of M3 isotopologues of aspartate (upper panel) and malate (lower panel) normalized to M5 aKG from [U-^{13}C]glutamine throughout pluripotency progression (n = 3). (**C**) Fractional ^{13}C incorporation from [U-^{13}C]glucose (G) and [U-^{13}C]glutamine (Q) into indicated

histone acetylated peptides in EpiSCs at the indicated time points. Residues separated by “l” indicate the presence of one residue or the other (n = 3). (**D**) Heatmap showing L/H ratios (where L=sample and H=internal standard) for specific histone post-translational modifications across ESCs, EpiLCs day 2, and EpiSCs. The data has been normalized to the average levels of the corresponding histone peptide measured in ESCs (n = 3). (**E**) Heatmap showing the normalized ^{13}C incorporation for differentially modified histone peptides derived from [U-^{13}C]glucose (Glc) or [U-^{13}C]glutamine (Gln) in ESCs, EpiLCs day 2 and EpiSCs. Residues separated by “l” indicate the presence of one residue or the other (n = 3). (**F**) Ratio of the glutamine to glucose ^{13}C-incorporation for the indicated histone post-translational modifications in ESCs, EpiLCs day 2 and EpiSCs (n = 3). (**G**) Representative whole-mount IF images of NANOG and H3K9me2 in E3.5 and E6.5 mouse embryos. Dotted line depicts the ICM (E3.5) and the epiblast (E6.5) respectively. Scale bar = 20μm (**H**) Relative fluorescent intensity of H3K9me2 normalized to DAPI. At least 6 embryos were quantified for each staining. (B) Data represent the mean of biological replicates ± standard error of the mean (SEM) per condition, with each colored point indicating an individual biological replicate. (C) Data represent the mean of biological replicates ± standard error of the mean (SEM) per condition. (F) Data represent the mean of biological replicates, with each colored point indicating an individual biological replicate. (B, F) Statistical significance was assessed using one-way ANOVA followed by Tukey's HSD post-hoc test. Levene's test was used to evaluate homogeneity of variances, and Shapiro-Wilk test was applied to assess the normality of residuals for each metabolite. (H) Statistical significance was assessed by Mann-Whitney unpaired test.

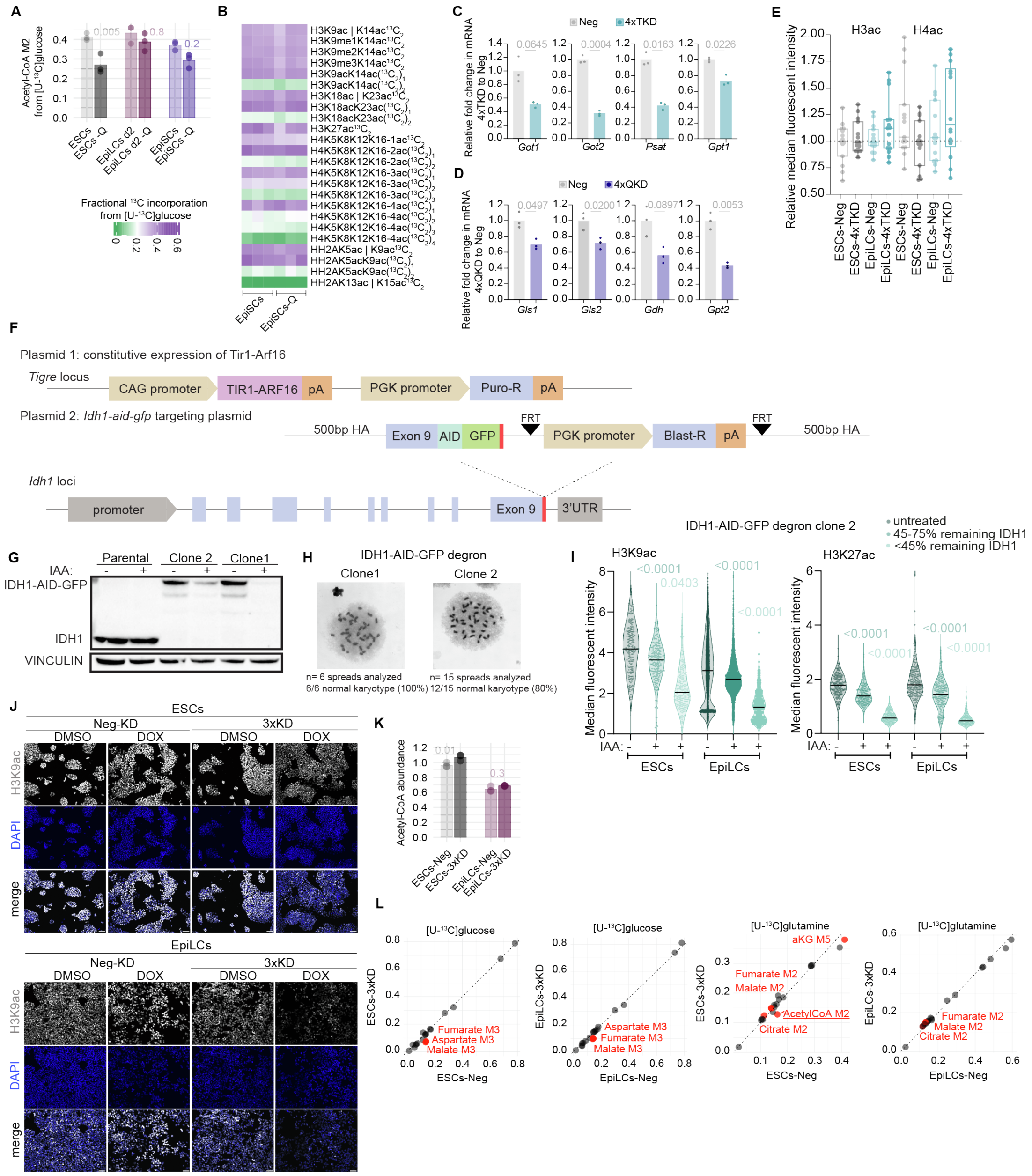

Supplementary Figure 6 – Glutamine metabolism is a source for histone acetylation - Related to Figure 6

(**A**) Fractional labeling of acetyl-CoA (M2 isotopologue) from [U-^{13}C]glucose in ESCs, EpiLCs and EpiSCs under glutamine-replete or deprived (-Q) conditions (ESCs, n = 2; ESCs -Q, n = 3; EpiLCs d2, n = 2; EpiLCs d2 -Q, n = 3; EpiSCs, n = 3; EpiSCs -Q, n = 3). (**B**) Heatmap showing the fractional ^{13}C incorporation of [U-^{13}C]glucose in the indicated histone acetylated peptides EpiSCs under glutamine-replete or deprived (-Q) conditions. Residues separated by "I" indicate the presence of one residue or the other (n = 3). (**C, D**) RT-qPCR analysis of the indicated genes in scramble control (Neg) and following the repression of *Got1, Got2, Psat* and *Gpt1* (4xTKD) or *Gls1, Gls2, Gdh* and *Gpt2* (4xQKD) under DMSO (gray) or DOX (blue or purple) conditions. mRNA levels were normalized to the housekeeping genes *Actin* and *Tbp.* (**E**) Relative median fluorescent intensity of indicated histone acetylation marks in Neg and 4xTKD cell lines in ESCs and EpiLCs day 2. For each histone acetylation mark, the fluorescent intensity was calculated by measuring the median intensity of DOX-treated cells normalized to their DMSO counterparts. Data corresponds to 5 biological

replicates (n>20.000 cells). Median, maximum and minimum values are indicated. (**F**) Plasmids employed for the generation of the E14 TIR1-ARF16; IDH1-AID-GFP ESC clones. Plasmid 1: Integrated into one *Tigre* allele for constitutive expression of the TIR1-ARF16 protein under the control of the CAG promoter. This plasmid also includes a puromycin resistance cassette driven by the PGK promoter. Plasmid 2: Targeting plasmid used for the integration of AID-GFP into the *Idh1* loci at the last coding exon (exon 9) using the appropriate homology arms (HA). This construct contains a floxed blasticidin resistance cassette driven by the PGK promoter. (**G**) Immunoblot analysis of whole-cell lysates from parental E14-WT and E14 TIR1-ARF16; IDH1-AID-GFP ESC clones untreated or following 24-hour treatment with IAA. Samples were probed with Idh1 and Vinculin antibodies. (**H**) Bright-field image of a chromosome spread used for karyotyping IDH1-AID-GFP clones. Analysis of 6 and 15 metaphase spreads from clone 1 and clone 2, respectively, revealed that 100% of clone-1 spreads and 80% of clone-2 spreads exhibited a normal karyotype of 40 chromosomes. (**I**) Median fluorescence intensity of the indicated histone acetylation marks in ESCs and EpiLCs (day 2) of IDH1-AID-GFP cells (clone 2). For each histone acetylation mark, fluorescence intensity was quantified under untreated control conditions and in IAA-treated (7.5h) cells stratified according to residual IDH1-AID-GFP levels (cells retaining 45–75% IDH1 and cells with <45% IDH1). Each dot represents a single cell, and data corresponds to five biological replicates (n > 800 cells). (**J**) Representative IF images of H3K9ac (gray) in Neg and 3xKD cell lines in ESCs and EpiLCs day 2 in the presence of DMSO or DOX for 72 hours, grown in the corresponding cell culture media. Scale bar = 50µm. (**K**) Acetyl-CoA abundance levels in scramble control (Neg) and pyruvate cycling-deficient (3xKD) ESCs and EpiLCs day 2. The values represent ion counts normalized to protein content (ESCs-Neg, n = 3; ESCs-3xKD, n = 3; EpiLCs-Neg, n = 3; EpiLCs-3xKD, n = 3). (**L**) Scatter plots comparing the fractional ^{13}C enrichment in TCA cycle intermediates from [U-^{13}C]glucose or [U-^{13}C]glutamine between scramble control (Neg) and pyruvate cycling-deficient (3xKD) cells in ESCs and EpiLCs day 2. Statistically significant isotopologues are depicted in red. (A, K) Data represent the mean of biological replicates, with each colored point indicating an individual biological replicate. (A, K, L) Statistical significance was assessed using one-way ANOVA followed by Tukey's HSD post-hoc test. Levene's test was used to evaluate homogeneity of variances, and Shapiro-Wilk test was applied to assess the normality of residuals for each metabolite. (C,D) Statistical significance was assessed using two-way ANOVA. Shapiro-Wilk test was used to evaluate residual normality. (E, I) Statistical significance was assessed by Kruskal-Wallis test followed by Dunn's post-hoc test, with p-values adjusted with the Benjamini-Hochberg (BH) correction method.
